# Planning ahead with children with life-limiting conditions and their families: development, implementation and evaluation of ‘*My Choices*’

**DOI:** 10.1186/1472-684X-12-5

**Published:** 2013-02-05

**Authors:** Jane Noyes, Richard P Hastings, Mary Lewis, Richard Hain, Virginia Bennett, Lucie Hobson, Llinos Haf Spencer

**Affiliations:** 1 Noreen Edwards Chair in Health Services Research and Child Health, School of Healthcare Sciences, Bangor University, Bangor, UK; 2 School of Psychology, Bangor University, Bangor, UK; 3 Royal United Hospital Bath NHS Trust, Bath, UK; 4 School of Medical Sciences, Bangor University, Bangor, UK; 5 School of Healthcare Sciences and Centre for Health-Related Research, Bangor University, Bangor, UK; 6 Paediatric Research Nurse, National Institute for Social Care and Health Research Clinical Research Collaboration, North Wales Research Network, North Wales, UK; 7 Centre for Health-Related Research, Fron Heulog, Bangor University, Bangor LL57 2EF, UK

**Keywords:** Children, Palliative care, Advance care planning, Nursing, Medicine, Evaluation

## Abstract

**Background:**

The United Kingdom has led the world in the development of children’s palliative care. Over the past two decades, the illness trajectories of children with life-limiting conditions have extended with new treatments and better home-based care. Future planning is a critically under-researched aspect of children’s palliative care globally. This paper describes the development, implementation and evaluation of innovative child and parent-held palliative care planning resources. The resources were designed to facilitate parent and child thinking and engagement in future planning, and to determine care preferences and preferred locations of care for children with life-limiting conditions from diagnosis onwards. These resources fill a significant gap in palliative care planning before the end-of-life phase.

**Methods:**

Drawing on contemporaneous research on producing evidence-based children’s health information, we collaborated with leading children’s not-for-profit organisations, parents, children, and professionals. A set of resources (*My Choices* booklets) were developed for parents and children and evaluated using interviews (parents, children, professionals) and questionnaires (professionals) and an open web-based consultation.

**Results:**

Parents and children responded in three ways: Some used the booklets to produce detailed written plans with clear outcomes and ideas about how best to achieve desired outcomes. Others preferred to use the booklet to help them think about potential options. Remaining parents found it difficult to think about the future and felt there was no point because they perceived there to be no suitable local services. Professionals varied in confidence in their ability to engage with families to plan ahead and identified many challenges that prevented them from doing so. Few families shared their plans with professionals. Parents and children have far stronger preferences for home-care than professionals.

**Conclusion:**

The *My Choices* booklets were revised in light of findings, have been endorsed by Together for Short Lives, and are free to download in English and Welsh for use by parents and young people globally. More work needs to be done to support families who are not yet receptive to planning ahead. Professionals would benefit from more training in person-centred approaches to future planning and additional communications skills to increase confidence and ability to engage with families to deliver sensitive palliative care planning.

## Background

The study reported here builds on seminal research undertaken by members of our group on the importance of high quality and age-appropriate children’s health information to support child-centred decision-making and choice in children’s healthcare [1-6]. Our previous research has reviewed current practice and provided evidence to inform future development of children’s health information in the National Health Service (NHS) in the United Kingdom, with relevance to global contexts [3-5]. Findings from these major studies [4,5] make a significant and new contribution to understanding the types and formats of health information likely to inspire children and young people to make decisions and exercise choice. Virtually no age-appropriate and child-centred information explaining different types of service options and pathways to accessing services (for example hospital versus home care), or explaining different treatment or care options was identified. A lack of resources specifically for disabled children and those with complex and palliative care needs was highlighted.

In a further large scale evaluation of the National Framework for Children’s Continuing Care in England, we developed and evaluated a decision-support tool for healthcare professionals and once again found that child and parent-held resources to support essential processes of care, choice and decision-making were absent [2]. ‘Children’s continuing care’ is defined as an individually-tailored package of care needed over an extended period of time for children with complex health needs, which arise because of disability, accident or illness including life-limiting or life-threatening conditions. Children and their parents being referred for assessment for continuing care packages were not provided with appropriate information or care planning tools to help with thinking about their preferred types of continuing healthcare support and options regarding locations of care in different scenarios [2].

In the current overarching study, we were funded by the National Institute for Social Care and Health Research (NISCHR) to undertake research to develop a novel evidence-informed commissioning framework for children’s palliative care services in Wales [6]. Other aspects of the overarching study included:

• Mapping currently available services, ascertaining numbers, primary diagnosis at death and locations of death from an audit of children’s death certificates;

• Secondary analysis of the Millennium Cohort Dataset to establish the prevalence of children with life-limiting conditions in the population, and

• Health economic study of current spend on children’s palliative care services, and estimated costs of providing all children with an option of receiving end-of-life care at home.

In addition, we needed to ascertain the views and perspectives of children, young people and their parents concerning their care and service choices and preferred locations of care. This essential ‘service user’ evidence fed into the commissioning framework and informed decision-making about service costs to present to commissioners. From our previous work in this area, we knew that high quality child-centred information and care planning resources were not widely available. These resources were considered by us to be a vital link to support a key process of care (future planning), and a critical success factor to developing a robust children’s palliative care commissioning framework for the NHS. Therefore, it was decided to develop a suite of child and parent-centred future care planning resources to help capture service user perspectives to inform the commissioning framework, and for subsequent use in routine care planning. We developed a set of resources called ‘*My Choices*’ and ‘*Choices for My Child*’ booklets, and a directory of key children’s palliative care terms and services. The booklets were developed in collaboration with representatives from the Royal Colleges of Paediatrics and Child Health and Nursing, and four leading children’s not-for-profit organisations: The Association for Children’s Palliative Care (ACT), Association of Children’s Hospices (ACH), Contact-a-Family, and Care Coordination Network UK (CCN UK), along with colleagues from the ‘Lifetime Service’ an award winning children’s community nursing and psychology service in Bath, UK. ACT and ACH amalgamated into a single charity ‘Together for Short Lives’ in 2011.

The purpose of this paper is to describe the development, implementation and evaluation of the innovative child and parent-held *My Choices* resources to facilitate thinking and engagement in the future care planning process.

### Children’s palliative care planning and policy context

Families with children with life-limiting conditions and complex disabilities require early and ongoing support with their child’s health and social care from diagnosis onwards, and help to minimise the wider impacts on the family. In a children’s context - this type of support is called ‘palliative care’, and some children need this ongoing care over decades [7,8]. In this context ‘children’ refers to children and young people from birth to under 19 years. The groups of conditions identified as possibly leading to palliative care of children and young people, are as follows [8]:

1. Life-limiting conditions where cure is possible but can fail (e.g., cancer);

2. Conditions which, though treated intensively over a period of time, inevitably lead to early death (e.g., cystic fibrosis);

3. Progressive conditions where treatment is palliative and often over many years (e.g., muscular dystrophy); and

4. Irreversible but non-progressive conditions giving rise to severe disability and sometimes premature death (e.g., disabilities following brain or spinal cord insult).

Along with other high income countries, the Department of Health (England) and Welsh Government have made children’s palliative care and support to families a priority [9,10]. Recent reviews, policies and service frameworks have been designed to overcome problems in the continuity and coordination of children’s complex, palliative and continuing care [11-18]. There has also been an emphasis in higher income countries on developing children’s palliative care clinical networks and an integrated system of palliative care to optimize service delivery and organisation [19-21].

Guidelines and palliative care pathways signpost the need for healthcare professionals to share information at key time points and to involve children in decision-making [19]. The few currently available children’s care planning tools are primarily used by healthcare professionals and focus on decision-making towards or at the end-of-life [4]. Published literature is not clear about the decision-making processes and preferred care choices of children with palliative care needs and their families before the end-of-life care phase [6,22]. A recent review by Doug *et al*. identified that the concept of palliative care is absent from care pathways and frameworks that guide the process of transition from children’s to adult services [23]. In summary, although there had been considerable investment in children’s palliative care guidance and service delivery, there remained a notable absence of child and parent-held resources to support future care planning and decision-making. We set out to rectify this situation using evidence-based principles.

### Aim

The aim of this aspect of a larger study [6] was to develop and evaluate the ‘*My Choices* booklets’ for use by parents and children to facilitate thinking and engagement with future care planning.

### Conceptual frameworks

#### Conceptual framework for the evaluation of integrated palliative care networks

**Figure 1:**
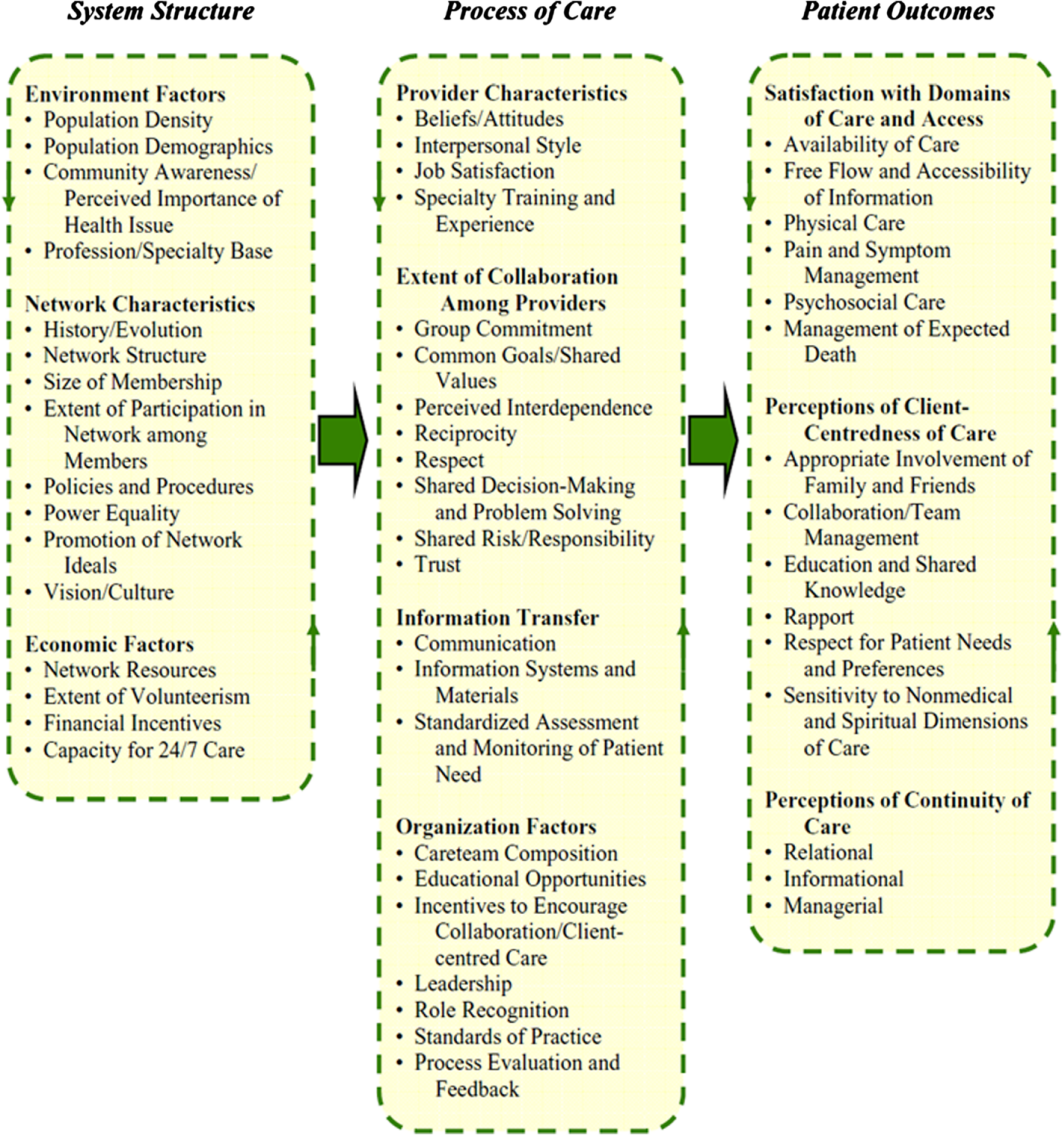
**Conceptual Framework for the Evaluation of Integrated Palliative Care Networks. **Copyright Bainbridge et al. BMC Palliative Care 2011. Reproduced with permission of Daryl Bainbridge and BMC Palliative Care.

Children’s palliative care is currently integrated and delivered by regional clinical networks. We used Bainbridge et al’s framework [21] to conceptualise the service delivery and organisation of children’s palliative care, within which child, family and client-centred care is a principal construct, information transfer and communication is a process of care domain, and key patient outcome domains include availability and access to care and the free flow and accessibility of information, and perceptions client-centredness of care such as shared knowledge and patient preferences (see Figure 1).

#### The lifetime framework

**Figure 2:**
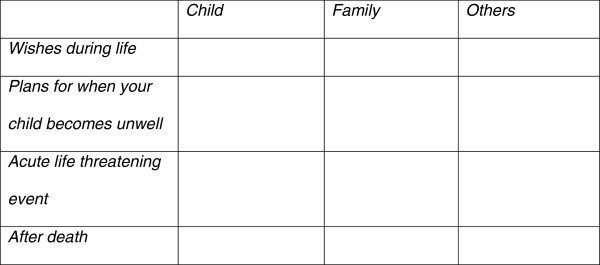
The Lifetime Framework for conceptualising care planning.

The Lifetime Service is an award winning children’s community nursing and psychology service, which has pioneered home-based care and support for children with non-malignant life-limiting illnesses and their families [22]. The Lifetime Framework is a ‘best-practice’ conceptual framework developed for use by healthcare professionals to structure their discussions with parents and, if appropriate, children. Its development is described in detail by Finlay et al. [22]. The original 3 × 3 framework includes the views of the child, family and ‘others’ involved before death, during an acute life-threatening event, at death, and after death (Figure 2).

#### Explanatory models of ‘partnership and participation’ in care and ‘translation of children’s health information resources into routine practice’

**Figure 3:**
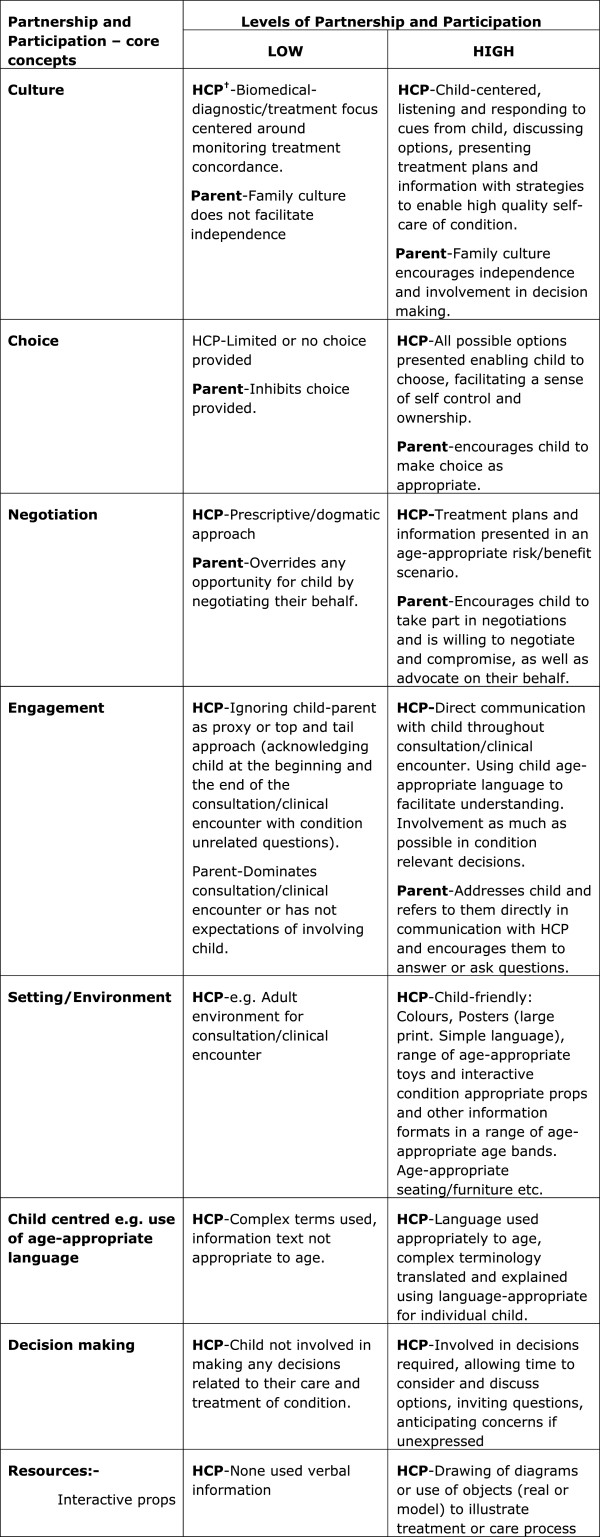
**Explanatory model of ‘Partnership and Participation’ between children, families and healthcare professionals in NHS contexts **[4]**.**

**Figure 4:**
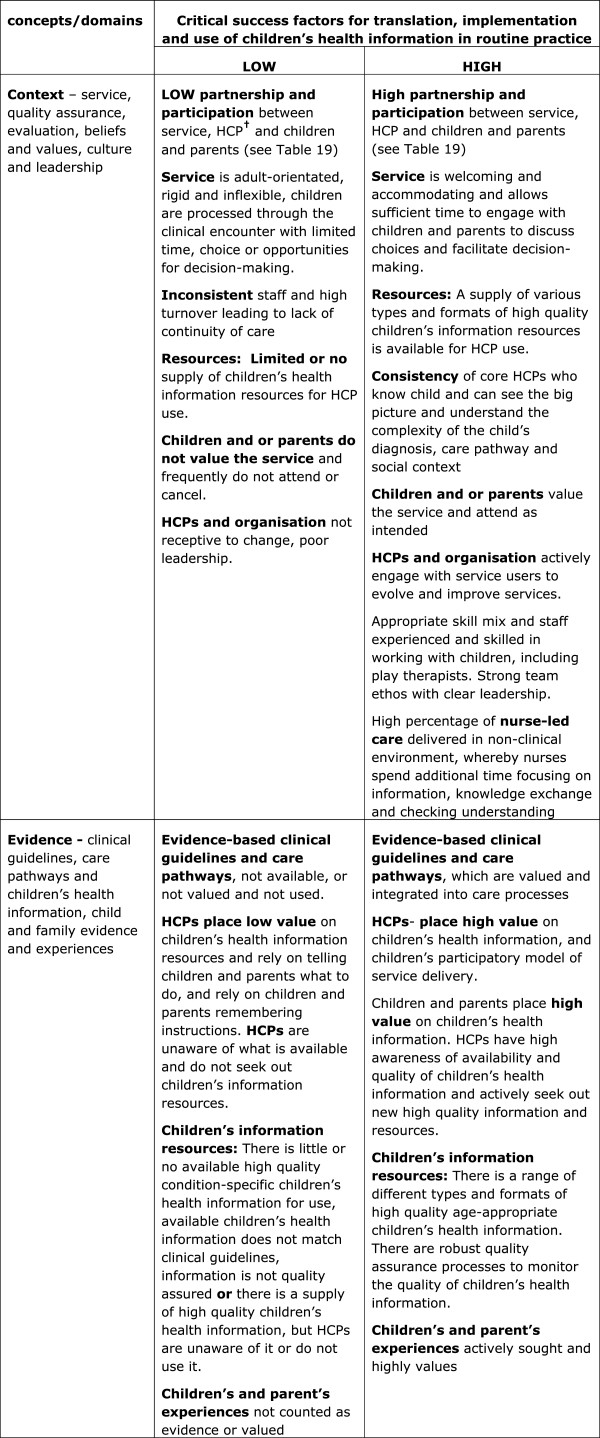
**Explanatory model of the critical success factors for translation, implementation and use of children’s health information in clinical practice by healthcare professionals **[4]**.**

We also used two explanatory models that were developed from the Children’s Health Information Matters Project [4]. One shows what high and low levels of ‘partnership and participation’ in care and decision-making between children, families and healthcare professionals looks like (Figure 3), and the second explains the critical factors associated with high and low levels of translation, implementation and use of children’s health information resources in routine practice (see Figure 4). These explanatory models show the importance of context in creating effective partnerships between families and healthcare professionals, the positive or negative value attributed to children’s information resources which dictates their use or not, and the role of active facilitation that is needed to successfully implement new interventions into practice. The models provide new clarity on the critical success factors for achieving successful partnerships with children and families in clinical encounters with healthcare professionals, and barriers and facilitators that need to be addressed for optimal implementation and use of children’s health information resources in decision-making.

## Methods

The following methods and processes were used to develop, implement and evaluate the *My Choices* booklets.

### Clarifying the focus and purpose of the ‘My Choices’ booklets

When we consulted with parents of children with life-limiting conditions in developing our project proposal, they gave us a clear steer that the care planning resources should focus on ‘living’ and not ‘end-of-life’. Parents said that in their experience children had many ‘near misses’ and could ‘bounce back’ to health again in unpredictable ways even if on a downwards spiral towards eventual death. Their children had also frequently outlived original medical projections of their life expectancy by many years, so there was a sense that parents wanted more control over how they planned ahead when things were uncertain and medical knowledge was lacking.

It was also apparent that parents would prefer a care planning resource primarily for their own use and, if appropriate, their children’s use and not one that looked like a medical or nursing care planning framework used by healthcare professionals to facilitate discussion with parents. Early discussions with parents suggested that a parent or child-held resource that ‘belonged’ to them could potentially act as a catalyst or object through which they could feel more comfortable thinking about and talking through sensitive issues, and could potentially be a source of empowerment. There are other exemplars – such as parent-held universal child health books that are liked and used by parents to record important information and trigger or facilitate timely discussion with healthcare professionals [24].

In parallel to our group developing the ‘*My Choices*’ resources for children and parents, Fraser et al. [25] were working to develop the ‘Wishes Document’ for healthcare professionals. This advance care planning documentation incorporates the Lifetime Framework and is designed to be used as a professional guide in response to family needs and requests. We considered a complementary parent and child-held resource to be important as most medical and nursing future care planning approaches require parents and, if appropriate, children and young people, to be prepared and receptive to engaging in the process and to be able to think through their needs and requests. We also liked the idea that if parents and children had an appropriate parent/child-centred ‘framework’ to organise and think through their ideas and preferences, they may potentially facilitate with greater confidence a conversation with healthcare professionals rather than the other way round, which according to parents we consulted is the way things currently generally happen in routine practice.

### ‘My Choices’ Booklet development

The diverse expert group that developed the initial booklets included: parent/young person representatives, a palliative care clinician, community nurse, children’s community physician, representatives from leading children’s not-for-profit organisations, authors of the Lifetime Framework, a psychologist, and children’s researchers. Drawing on evidence from the Children’s Health Information Matters Project [4], we produced an initial set of parent and age-appropriate child-held resources for the following groups:

• Boys 6–10 years;

•Girls 6–10 years;

• Boys 11–15 years;

• Girls 11–15 years;

• Young person over 16 years, and

• Parents

In addition, consultation with parents raised the need to produce a directory of key terminology used by healthcare professionals, and a description of the range of services, so that parents had access to relevant supporting information to engage with the planning process.

### Content of booklets

The overarching programme theory of the booklets (what the booklets were intended to do) was to help children, young people in age-appropriate ways, and parents, to:

• Think about their care now and in the future;

• Consider care choices and preferred locations of care in different scenarios;

• Facilitate discussion within families, and with healthcare professionals, and

• Keep a record that can be added to over time.

The booklets were designed to be used in a number of ways (programme logic), such as:

• At home and in private to facilitate thinking and help clarify thoughts and feelings and preferred care options; and

• During clinical encounters with healthcare professionals as a basis for sharing thoughts and information to inform care planning.

We had no preconceived ideas as to whether the booklets should be filled in or not, or merely used as a basis for thinking and initiating conversations.

The booklets followed the same basic format, with each version being tailored for the reader in age-appropriate ways, with the young people’s and children’s versions having less detail and content:

• Care at home and planning ahead

• Fun things/activities and future plans

• Care at school and planning ahead

• Staying well – planning ahead routine health checks and screening

• Growing up and moving to adult services

• Extent to which young person/parent is in control of future care planning processes

•“ What if” scenarios –

• What if parents need a short break?

• What if child/young person is not well? (divided into mild and moderately unwell for adult versions)

• What if child/young person is very unwell?

• What if doctors consider I/my child may not recover? (for adult versions only)

• Other important things to think about and plan.

The underpinning structure was adapted from the parent/child domains of the Lifetime Framework and, with permission, we incorporated a format for identifying and prioritising outcomes from a positively evaluated disabled children’s outcomes framework [26].

A teenage graphic design student (Victoria Hulme) produced age and gender appropriate images for the front covers. Based on evidence from the Children’s Health Information Matter’s Project [4] that children and young people wanted ‘realistic’ looking images, Victoria produced life-like colour images of different ages of children and young people. Early drafts were circulated to the expert group and partner not-for-profit organisations to gain feedback from their contacts (young people, parents, advocates). Revisions to images, colours, style and content were made according to feedback received.

### Mixed-method implementation and evaluation of the booklets with parents, young people, children and professionals

We drew on methods of theory-based implementation and evaluation and adopted both Pawson and Tilley [27], and Weiss’ [28] similar positions on mechanisms of action as summarised by Asbury and Leeuw [29] that ‘Interventions work (have successful ‘outcomes’) only in so far as they introduce appropriate ideas and opportunities (‘mechanisms’) for people (children, parents, professionals) in the appropriate social and cultural conditions (‘contexts’). The mechanism of change is not the intervention (*My Choices* booklets), but the behavioural response that the intervention and associated activities generate’.

### Evaluation questions

1. What do parents, children, young people and professionals think of the ‘*My Choices*’ booklets?

2. How have the *My Choices* booklets been used?

3. In what ways can the ‘My Choices’ Booklets be improved?

### Setting

Children’s complex health and palliative care NHS and social services, and not-for-profit organisations in North Wales.

### Participants

Children and young people with complex health and palliative care needs, parents, and multi-agency palliative care professionals.

### Implementation strategies

#### Network events with professionals in North Wales

To familiarise local professionals we introduced and presented the ‘*My Choices*’ suite of resources during three launch events and two local children’s palliative care professional network events. During these events we showcased the booklets and talked about the important role of healthcare professionals in facilitating their use if parents or young people chose to use the booklets during routine clinical encounters.

#### Distribution of ‘My Choices’ Booklets to parents, young people and children

Bilingual (Welsh/English) ‘*My Choices*’ booklets and study information packs were supplied to local participating children’s health and not-for-profit sector services that cared for children with complex health and palliative care needs. Given the sensitive nature of children’s complex healthcare and future care planning, local staff working within data protection principles identified parents who may potentially be interested in participating. Local staff adopted a variety of flexible approaches to distributing packs either in person, preceded by a telephone call, or through the mail. In line with all evaluations concerning sensitive topics and with vulnerable groups, this study will inevitably be subject to staff selection bias.

#### National network event

JN delivered a keynote presentation at a UK children’s palliative care conference, shared the ‘*My Choices*’ project website address, and invited professionals to forward information about the web-based consultation to parents and young people to gain their feedback on the ‘*My Choices*’ booklets. Copies of booklets were also shared with delegates.

#### Web-based distribution and consultation

*My Choices* booklets were made freely available for anyone to download from the project website. Partner not-for-profit organisations placed advertisements in their newsletters and on their websites inviting parents and young people over 16 years to visit the ‘*My Choices*’ project website, download and leave comments on the booklets, and if appropriate complete a booklet for research purposes.

### Data collection methods

We included an element of evaluation of the *My Choices* booklets in each of the following data collection methods used in the overarching study to develop a children’s palliative care commissioning framework [6]:

#### Semi-structured interviews with parents and young people and professionals

We adopted a generic qualitative approach [30] using semi-structured interviews to collect parents’, young people’s and professionals’ views on the *My Choices* booklets after distribution to parents via local services. Interview schedules were developed for different audiences, including parent, child, young person, and professional. Part of the interview focused on the *My Choices* booklets. Interviews were conducted at a mutually convenient time and at a location of the participants’ choice. With consent, interviews were recorded and we took digital photographs of examples of completed booklets, which were anonymised.

#### Pre-and post study questionnaire with professionals from participating services

Professionals were invited to complete an online or paper version of an anonymised pre-and post study questionnaire that requested feedback on the suite of ‘*My Choices*’ booklets. We provided participants with a number of items to be rated on a 5 point likert-type scale to ascertain their confidence in managing different aspects of children’s palliative care, and their views on the best places to care for children in specific scenarios. There were also opportunities to provide free text comments. We set up web-access to the online questionnaire via laptop computers at launch and professional network events, or professionals could access and complete the online or paper version at their own convenience. We issued each participant with a unique code, and after the *My Choices* booklets had been in circulation for 6 months we asked those that had completed a pre study questionnaire to complete a follow-up post study questionnaire.

#### Web-based consultation

We designed a brief optional online survey to capture general feedback from people downloading the *My Choices* Booklets from the website. The consultation was open for the duration of the study until the final report was submitted (2008–2011).

### Data analysis

#### Qualitative interviews

Interviews were transcribed verbatim and managed using Atlas Ti software. Ritchie and Spencer’s five step Framework approach to applied policy data analysis was used to guide analysis [31]. The Framework approach is particularly suitable for policy-orientated studies that specify clear policy aims and questions at the outset. The five steps are as follows:

1. Familiarisation. Transcripts were scrutinised by the core team (VB, LH, LHS, JN) to get a feel for the entire dataset and for significant emerging themes.

2. Identifying a thematic framework. Key emerging concepts, constructs and themes that reflected the aims and research questions were transformed into an index of codes.

3. Indexing. The preliminary coding framework of index codes was agreed and applied to transcripts using qualitative data analysis software Atlas Ti [32].

4. Charting. We produced tables to compare coded data across cases and between professional groups.

5. Mapping and Interpretation. Charts and field notes were reviewed by the team to look for patterns emerging across the dataset and associations within it.

#### Descriptive questionnaire data

Questionnaire data were analysed with descriptive statistics using SPSS. Open ended responses were extracted into a table, grouped and subject to content analysis [33].

#### Web-based feedback

Responses were summarized using survey monkey [34] and the free text responses were collated.

### Ethical issues

Approval was granted from Bangor University and local NHS ethics committees. Written consent was obtained from participants over 16 years, written parental consent, in addition to child assent was obtained for children under 16 years. Data were anonymised or redacted.

### Sample

#### Parents, children and young people participating in interviews

We adopted a convenience sampling approach, whereby we aimed to interview those who returned their contact sheet, with a target sample of up to 20 parents and 20 young people. This group of children are, however, prone to sudden illness and deterioration in their condition. Sadly, one young person who consented to participate died suddenly prior to interview. Two other families who expressed a willingness to participate were not able to do so as their children subsequently became acutely unwell.

We interviewed 12 parents and 3 bereaved parents (11 mothers and 4 fathers) who cared for children and young people with complex healthcare and palliative care needs, and 11 children and young people from 10 families (whose participation varied from active (3) to passive (8) depending on their impairments). Where appropriate, parents conveyed the experiences and choices of children and young people with profound sensory and communication impairments.

As these parents and children are easily identifiable due to their family circumstances and children’s relatively rare diagnoses, we have listed broad demographic/diagnostic categories of 11 index children/young people of 13 parents (excluding the children of 3 bereaved parents) in Table 1.

**Table 1:** Broad demographic categories of 11 children and young people

**AGE**	**RCPCH/ACT GROUP **[8]	**GENDER**
School age	2	M
School age	1	M
Pre school	4	M
Pre school	4	F
School age	4	F
Young person	4	M
Pre school	2	F
Young person	2	F
Young person	1	F
Young person	4	M
Young person	4	F

KEY: 0- <5 years = pre-school.

>5-15 years= school age.

16 years and above = young person.

### Professionals

#### Semi-structured interviews

We purposively selected a range of health and social palliative care professionals who expressed a willingness to participate in an interview. We aimed to recruit 10, and interviewed 13. Professions represented in the sample included: community children’s nurse, hospital doctor, community doctor, physiotherapist, school nurse, social worker, and psychologist.

#### Questionnaire with professionals

Twenty-seven completed the pre-study questionnaire and twenty of the original respondents (74%) returned a follow-up questionnaire. We estimate that the sample represents around half of those professionals who have a significant focus on children’s palliative care in the study region.

#### Response to web consultation

The response to the web consultation was disappointing and did not match with partner not-for-profit organisation expectations. Only two parents completed the booklets online and completed the optional survey, and so this evidence is included with interview data below.

With hindsight, we should have inserted a traffic monitor to the website to ascertain the number of hits and downloads. Booklets downloaded from the website had a DRAFT watermark on every page. Anecdotally, we became aware at dissemination events that healthcare professionals from outside of the study region had accessed draft booklets via the study website, but had not left feedback or completed the optional survey, and in the absence of other appropriate resources had already begun to adapt the draft booklets for local use. On an unrelated visit to a children’s community nursing service in England, healthcare professionals were found to be working with draft *My Choices* booklets, thereby reinforcing the need to produce and evaluate high quality children’s palliative care information resources.

## Findings

When evidence from young people and parents is mapped against the conceptual framework for integrated palliative care (Figure 1), the overall picture reveals incomplete local children’s palliative care service provision with important gaps in the network [21]. For example, families generally reported limitations in the availability and access to care, and wide variation in the accessibility of information, lack of shared knowledge and planned elicitation of child and family care preferences and preferred locations of care in different scenarios (i.e. context). The ‘context’ as described reinforced the need for the wider study to develop a commissioning framework to better meet the needs of children with palliative care need in this region of Wales. Views and perceptions of the *My Choices* booklets need to be located in this wider service delivery context to interpret and make sense of the evidence.

We identified several different mechanisms of action (behavioural responses to the *My Choices* booklets). For some parents and young people the *My Choices* booklets did stimulate thinking about care now and in the future – but not always in the way as originally intended (programme theory and logic). The *My Choices* booklets were almost exclusively used in private at home by parents and young people. During the time (up to 6 months) that parents and young people were in possession of the *My Choices* booklets prior to being interviewed, there was minimal sharing of booklets, thoughts, information or ideas with healthcare professions.

### Use of the My Choices booklets

Parents and young people roughly fell into three groups. Those that liked the booklets and felt that they could usefully use them to record information (mechanism 1), those that were positive about the purpose of the booklets as a framework for thinking about care options but did not necessarily want to record information in them (mechanism 2), and those that did not feel able to think about the future or future care planning, or were cynical as to whether the NHS would be responsive to their plans and ideas about how best to manage their child’s care (mechanism 3).

For young people and parents who were sceptical of the benefits of person-centred planning - the entire context (culture and ethos and experience of service delivery) would need to change for optimal implementation of the ‘*My Choices*’ booklets and positive engagement with decision-making to occur. When mapped against the core concepts of the partnership and participating in decision-making explanatory model (Figure 2), it was clear that these specific young people and their parents experienced very low levels of partnership and participation due to the culture of their State-provided services that did not empower families to decide for themselves: there was a lack of choices offered, they experienced a lack of negotiation, engagement and child-centredness of the system, as well as lack of resources in the system. Implementation of a future child and parent-held care planning framework alone cannot mitigate for weaknesses in context elsewhere in the system. For example, one parent commented:

‘Well, I remember reading this (My Choices booklet) and I thought “why the hell should we do this? We wouldn’t get anything anyway!” Ha ha ha!’

(Mother of primary school age child a)

In line with the framework for integrated palliative care (Figure 1), a current policy aspiration is for people to have more choice and control over their care and ‘child/person-centred planning’ is a key commitment to NHS users [11-19]. An adult teenage girl thought that the booklet and planning for the future was a good idea – especially if available on-line, but felt that other people did not always agree with what the young person wanted. She also felt that services needed to change the way they worked before person-centred care planning could benefit her. She explained:

‘So it would be good in a way, but in other ways, people would have different opinions of it don’t they, and go against stuff so….’

The mother of an adult teenage girl went on to explain:

‘I think it’s a lovely thing if someone took it on-board, brilliant - I think the whole service needs to alter to be able to incorporate something like that.’

(Mother of adult teenage girl a)

Parents were also very sceptical that professionals would want to listen to them, or had any additional resources to change or individually-tailor existing care provision. The following experiences of two mothers were common:

‘I wouldn’t, say, go to social services and have a read of that, because no matter what you say, they don’t listen’.

(Mother of primary school aged child a)

‘… and I don’t know who would listen to it really. Because I think services we have at the moment, are doing what they can under the duress that they have to, like I say, if I have a problem with this…. but I don’t think that people could help anymore than they’re helping.’

(Mother of adult teenage girl a)

Parents who were less positive about the booklets also lacked clarity on the purpose of the booklets, and sometimes confused them with ‘assessments’ and application forms to be completed to gain access to a service. Parents of children with highly complex needs are used to eligibility criteria of services and resources based on pre-determined levels of need and disability, so perhaps rather wisely, parents were wary and weary of filling in forms and generally preferred not to write things down or complete the booklets.

Parents who were receptive to planning ahead had thus far mostly preferred to use the *My Choices* booklets as a way of raising their own awareness about care planning and organising their thoughts. Some parents had, however, already used the booklets – for example, thinking about planning for their child’s transition to adult services. When parents did complete the booklets and gave us permission to photograph anomymised pages, they had used the booklet as anticipated and developed a clear plan and rationale for what would improve their situation or meet their needs in different scenarios (see Additional file 1). Many of the issues that parents identified (such as help to care for their child at home when unwell to avoid hospitalisation) had clear and practical solutions documented (for example – flexible and responsive community children’s nursing input when child unwell). Parents also documented that they preferred things like blood samples to be collected from their child at home, and interventions such as intravenous antibiotics to be administered at home. These parents also talked about using their booklets over time as their perspectives on preferred locations of care could change as their child’s needs changed. Parents’ plans and ideas were consistent with current policy aspirations to improve outcomes for children with complex healthcare and palliative care needs (such as delivery of care in location of choice).

When mapped against the critical success factors in the explanatory model for translation of children’s health information in routine practice, it was clear that the context of clinical encounters needed to be strengthened further to enable forward planning to happen. We observed that healthcare professionals had been hindered in their efforts to facilitate forward planning due to lack of resources such as the *My Choices* booklets, and they needed additional support to increase their communication skills in children’s palliative care contexts to proactively facilitate the sensitive conversations needed to forward plan with parents and children.

Nurses acknowledged that the *My Choices* booklets could be valuable for working with children and young people. A community children’s nurse perspective was typical:

‘… with the children with complex health care needs, most of them have significant associated learning difficulties. And you know, I think this is a really useful thing for somebody to use as a prompt and aid memoir to work with children [who are able to engage with care planning], around erm, what, what, to cover some difficult subject areas’.

(Community children’s nurse)

### Transition to adult services

One family for whom transition from children’s to adult services was a relevant issue reported that they had only started considering future care options when looking through the *My Choices* booklet in their preparation for their research interview. The booklet initiated a fearful and frustrated response in the parent:

‘…. *another thing that does quite annoy me is*, *everything*, (*name of child*) *is in a category*, *but* (*name of child*) *is an individual*. *So* (*name of child*) *is coming up to adulthood*, *but she*’*s still a baby*, *and what my fear is*, *and I*’*ve got this in my head now*, *when she reaches 18*, *the respite ceases so this is why I*’*m looking now*, ‘*cos when I think of* (*name of child*), *I*’*ve got to say*, *leaving school*, *on a Friday*, *coming home*, *24*/*7*, *give me a noose and I put my head in it*. *I*’*m being really honest*’.

(Mother of secondary school age teenage girl)

In the absence of a care planning resource such as the *My Choices* booklet, information about transition and care options had mostly been gathered independently by parents. Where possible parents involved their children in decisions to achieve the best possible outcomes:

‘What we want to plan for (name of child), it’s for him to be able to - not to just be reliant on…, it’s to be his choice and what he wants to do, where he wants to go. And not just, you know, it’s really important for him to have his funding and there’s all that, and that’s critical. Because that will give him choices in life’.

(Mother)

The young people also talked about the importance of having a simple resource to record plans for transition to adult services. A teenage girl said:

.. ‘*At least then*, *I would know what to expect from it*’.

(Adult teenage girl a)

Her mother also found the booklet format and information useful:

‘*I think it focuses all the points*. *I think we know how we feel on all of them*…’

(Mother of adult teenage girl a)

### Signposting to additional information

Access to additional information was also considered beneficial by parents. The mother of another teenager said:

‘*All info is good info*, *we*’*ve found it before*, *sometimes you can*’*t get the answers as you want because you*’*re not asking the right questions*, *so if you give*, *regardless of what it is*, *if you read something and even if you just pick a couple of paragraphs out that means something to you at that time*, *you know what I mean*? *Or further down the road you*’*ve read it*, *and then further down the road*, *it*’*s relevant*, *so all info is good info you know what I mean*?’

(Mother of adult teenage girl b)

### When a child dies

A social worker described the *My Choices* booklets as a valuable resource for the family and siblings after the death of a child. A booklet for siblings could also be long term: less of a care planning framework for the disabled child, and more like a memory book and diary for organising their thoughts and documenting their preferences for what happens to them when their sibling is unwell or requires respite:

‘*Some of the things that they talked about before the death*, *they might need to re*-*visit and that booklet might be very important*, *about erm*, *as a memory of what the child had wanted or*, *and when they reflect back they can say*, *the best thing for us*, *is that we know we got it right*, *because we talked about this*, *and this is the document of when we talked about it*.’

‘.. *And I guess as siblings get older*, *if they were confused about any aspects of care*, *those books would become like a very special kind of family memento*, *it might help the siblings if there was any confusion about what had happened*, *you know if they said* “*but*, *how do you know that*, *that was done right*?”, *they could say* “*well*, *look*, *we wrote this down*, *at this point*, *this here*, *that*’*s what we did and that*’*s how we knew what to do*’.

(Social worker)

### Preferred locations of care

When working through the booklets, parents and young people consistently indicated to the researcher that whenever possible they wanted to be looked after at home, with hospital being a last resort. Additional short break care was appreciated by some families away from the home (for example, the children’s hospice). In contrast, professionals were far more ambivalent about care at home if the child became unwell. Around half of professionals felt that children with serious illness should be cared for at home, whereas parents told us that they rarely called an emergency ambulance even if their child’s condition sometimes merited it.

### Sharing of information between parents, young people and professionals

At the outset of the study we were interested to know if parents and young people would share (or not) their own *My Choices* care planning booklets with healthcare professionals. Findings from the 20 professionals who responded to the post study questionnaire revealed that only one reported parents or children/young people had “once or twice” shared their filled in *My Choices* booklet with them. This lack of sharing information matches with parents’ narratives about the booklet being theirs and to help them think about things, rather than share the content with others. Six months also may not have been sufficient time for parents to start thinking about whether they wanted to, or how best to use the booklet, or whether there were significant care planning issues that they felt needed their attention during this relatively brief time. In addition, some parents may not have met with their healthcare professionals since receiving the booklets.

Those healthcare professionals who felt that the *My Choices* booklets would be helpful, also suggested that the content could be photocopied and kept within the service as a shared resource.

‘Definitely, yeah, I mean it’s, the idea of it is great isn’t it? …. something like that, if you could duplicate once it’s been completed, then they could have a copy on the ward, erm, because they don’t know how to look after these children, on the ward.’

(Community Nurse)

### Previous parental experiences of care planning

Evidence from families who had been involved previously in care planning indicated that there was no consistent approach locally or nationally. Care planning was often dictated by parents following a change in their child’s condition. There was some evidence of planning ahead but this was often only for short periods for example, for an hour a day with hands on care, during summer holidays and frequently this additional care was unavailable. Parents were also worried about planning too far ahead as their child’s condition could change. The following mother described her experiences of care planning:

“*No*, *we do just six months at a time*, *because I think*, *you know*, *I sort of like tend to look at the here and now*, *because this is to me what*’*s important*, *what*’*s happening now*. *You know*? *Twelve months time*, *something totally different could happen*, *and so I just think*, *right*, *if we deal with now*, *rather than worry about twelve months time*, *and I can think about that when it comes*..’

(Mother of pre-school and primary school aged children)

Some parents indicated that they had not been involved in planning future care before now:

‘Interviewer: ‘… so we’ve been … thinking about the future, and whether anybody has ever shown you anything like an advance care plan or pathway? [mother indicating no] Nobody has ever discussed any of those?’

Mother: ‘No’

(Mother of primary school aged child b)

### Professional experiences of care planning

**Figure 5:**
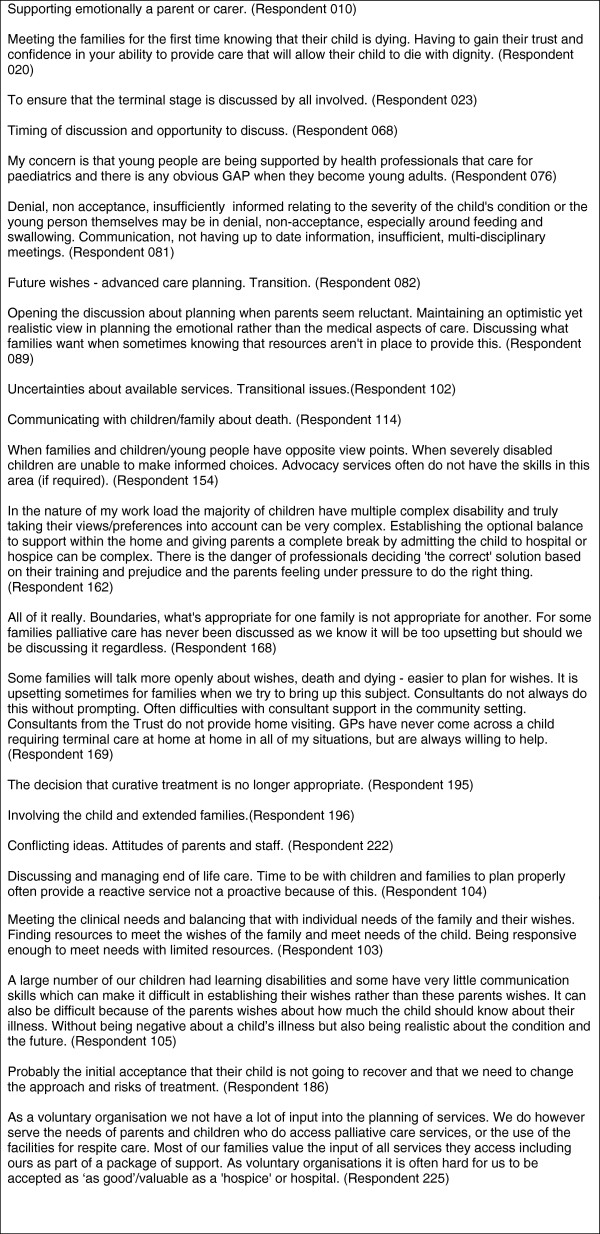
Aspects of palliative care planning with children/young people and their families that professionals find most challenging.

Responses to questionnaires indicated a distribution of confidence in care planning across the board with an equal spread across all responses from those who were confident and engaged with families to those who were not at all confident and did not feel able to actively engage with families. In addition to not wanting to raise unrealistic expectations, interview, questionnaire and free text responses indicate that many healthcare professionals find future care planning and raising sensitive issues with children and families challenging, and they were concerned that they got it right. For example, in free text responses returned with questionnaires, it was evident that healthcare professionals also struggled with a multitude of personal, professional and resource barriers that prevented them from engaging in future care planning with families. Healthcare professionals found it difficult to engage with and support children and parents, especially towards the end-of-life and in circumstances where understanding was not shared between professionals and families. Young people were acknowledged as having different views to their parents and healthcare professionals found it challenging to deal with these differences. ‘How to’ start the process of future and transition planning and how to do it effectively when expectations did not match actual service provision were consistent concerns. Responses are summarised in Figure 5.

### Style and format of the My Choices Booklets

One of the adult teenage girls interviewed liked the My Choices Booklet and filled it in, but did not have any suggestions for change to the style or format.

Parents expressed a wide variety of opinions about the style and format, with different things suiting different parents. For example, one parent hated the rating scale to determine how important it was to change an aspect of care, whereas others liked the rating scale. Overall we received useful feedback about the need to reduce and simplify further some of the content and page layout.

Of the 12 professionals who provided feedback on the booklets, the majority found them clear and easy to understand, although there was a wide range of views on developing the content further. Overall, just over half of the professionals (most of whom had not yet used the booklets with parents) rated them as moderately or very helpful for professionals and families.

Both professionals and parents were concerned that we had not produced a *My Choices* booklet for siblings. We acknowledge this as a gap and will anticipate producing a sibling booklet in the future. One mother’s comments were typical:

‘I think a child (sibling) booklet would be brilliant, start directly from the beginning, go through (name of child’s) life, and you know what’s affected him, what he looks at (name of child) now, and what he looks at (name of child) in the future. And erm, how would (name of child) see himself?

(Mother of primary school aged child c)

Most parents were conversant with all terminology used in the booklets, whereas one mother did not know what a key worker was – despite the fact the she should have access to one to co-ordinate her child’s care. (A key worker is a person assigned to a family to facilitate child and family-centred planning and co-ordination of care on their behalf). Another parent felt that we had spent too much money and created an expensive suite of resources, when a less fancy resource would be sufficient for parents and children to use. We took this as positive feedback as we had produced the booklets ourselves using desktop publishing software, without any significant resources.

## Discussion

The number of children with palliative care needs is increasing [8,35-37]. As illness trajectories increase, families and professionals need to be receptive and open to consider planning for a range of different care scenarios individual to each child and family [22]. Planning ahead needs to be undertaken on an ongoing basis as the child and family’s circumstances change over time – especially around transition to adult services [8]. The *My Choices* booklets provide an individually-tailored framework that young people and families can choose to use if helpful. In this evaluation the *My Choices* booklets appeared to most enable those young people and their parents who were receptive and able to think about how their care could be better managed, and helped them to consider options as to where they would potentially like to be cared for in different scenarios. Challenges remain in providing an appropriate context whereby children and families feel empowered to routinely share their thoughts and ideas with professionals.

Greater understanding of how parents and especially mothers use hand-held resources with professionals comes from recent longitudinal evaluations of universal hand-held child health and maternity books [24]. Key critical factors for their successful use included parents valuing the book and its purpose, and healthcare professionals being familiar with the resource and using an appropriate facilitative style and tone when information was shared and recorded [24]. In the current study, parents and professionals did not always value the *My Choices* booklet because of their knowledge of service limitations and barriers to accessing services, or perceived challenges in communication with professionals, or their children. Until these issues are addressed, it is unlikely that person-centred future planning can or will be realized.

Since the introduction of hand-held records, public health nurses and midwives have identified the need for specific training to help them develop and adopt appropriate models of parent participation in shared care planning. Experience from the current study suggests that staff and parents would benefit from additional training and support to actively engage with a future care planning resource such as the *My Choices* suite of booklets.

### Strengths and limitations of study

Strengths of this study include the user-centred and evidence-based development of the future planning resources with key not-for-profit organisations, and the history of evidence-based development of the lifetime service and Framework, upon which the My Choices booklets were based. Limitations include the relatively small qualitative sample for evaluation, and the lack of sharing of information generated by use of the My Choices booklets by parents and young people with healthcare professionals over the 6 month evaluation period. Six months may be a too short time period to see this type of behaviour change. Nonetheless, the study fills a gap in knowledge and novel findings are likely to be transferrable to other palliative care contexts.

## Conclusion

The innovative *My Choices* booklets, fill a significant gap in future care planning tools before the acute end-of-life phase. Following evaluation and further post hoc revision, the booklets and have been endorsed by Together for Short Lives are now free to download and adapt for local routine use by children, parents, the NHS, third sector, and health organisations globally. Local adaptation could include adding appropriate contextual pictures and artwork. For copyright reasons we have removed artwork produced for a local Welsh context. Versions are available in English (see Additional file 1, Additional file 2, Additional file 3, Additional file 4, Additional file 5, Additional file 6, Additional file 7 and Additional file 8), and Welsh see [6].

Parents’ and young peoples’ ideas and individually-tailored plans for care generally match current children’s palliative care policy aspirations. For some parents and young people, the *My Choices* booklets were a helpful resource and provided clear evidence regarding preferred locations of care and models of service delivery to inform the commissioning framework for services [6].

More work needs to be done to understand and support parents who are not at all receptive to thinking about care preferences. Greater understanding is needed concerning how young people’s and parents’ preferences change over time and to what extent actual real time decisions are based on availability of resources or lack of flexibility and responsiveness of overstretched services.

## Competing interests

The authors declare that they have no competing interest.

## Authors’ contributions

JN designed the study with RHastings. ML and RHain advised on booklet development. VB and LH collected, analysed and interpreted data with support from LHS. JN drafted the manuscript with all authors providing critical review and final approval.

## Authors’ information

JN has experience in child health research, health services research and health economics and evidence synthesis.

RHastings specialises in research with disabled children and adults and their families.

ML led development of the award winning Lifetime Service in the UK, and has experience of commissioning and evaluating children’s services as an Executive Nurse.

VB has experience in delivering child health services and is currently a nurse educator.

LH has experience working as a Children’s Community Nurse in Palliative Care and is currently a Paediatric Research Nurse.

LHS has experience in educational and child health research, and is currently a research officer.

RHain Honorary Senior Lecturer, Bangor University, Consultant and Lead Clinician Paediatric Palliative Care Children’s Hospital, Cardiff, UK.

## Pre-publication history

The pre-publication history for this paper can be accessed here:

http://www.biomedcentral.com/1472-684X/12/5/prepub

## Supplementary Material

Additional file 1 **Photographs of completed My Choices booklets. **Illustration of completed My Choices booklet. Click here for file

Additional file 2 ** parent booklet 2012. **Blank booklet to download and use. Click here for file

Additional file 3 **My Choices Young person age 16+ 2012. **Blank booklet to download and use. Click here for file

Additional file 4 **Children’s complex healthcare UK service directory 2012. **Complex health key terms and directory for download and use. Click here for file

Additional file 5 **My Choices 6–10 years boy 2012. **Blank booklet to download and use. Click here for file

Additional file 6 **My Choices 6–10 years girl 2012. **Blank booklet to download and use. Click here for file

Additional file 7 **My Choices 11–15 years boy 2012. **Blank booklet to download and use. Click here for file

Additional file 8 **My Choices 11–15 years girl 2012. **Blank booklet to download and use. Click here for file
