# Supplementary material for: Planning ahead with children with life-limiting conditions and their families: development, implementation and evaluation of ‘My Choices’
Source: BMC Palliat Care. 2013 Feb 5;12:5. doi: 10.1186/1472-684X-12-5 (PMC3579717; doi:10.1186/1472-684X-12-5)
Supplement: Additional file 1 — Photographs of completed My Choices booklets. Illustration of completed My Choices booklet. [file 1472-684X-12-5-S1.pdf]

Caring for my child every day at home.

Parents frequently need practical help and support to care for their children with complex healthcare needs and disabilities at home.

Is this an aspect of your child's care that you want to change or improve?
If yes, please provide a brief description of your current situation

YES MORE CARE IN THE HOME AS WE HAVE NONE AT THIS TIME

Do you have a 'key worker' to coordinate care? *Circle* **Yes** **(No)** **Don't know**
If yes, please describe (e.g. their name, their job title, what they do)

What works well at the moment with your child's everyday care?

WE CAN ACCESS CHIC NURSE DANNA NURSE HOSPITAL & DR VERY WELL

What would you like to work better at the moment with your child's everyday care?

HELP DAY TO DAY IS NEEDED IN THE HOUSE

How important is it to change/improve this aspect of your child's care?
Circle one option

(High priority) **Medium priority** **Low priority**

Follow-up appointments and routine tests

Children with complex healthcare needs and disabilities frequently attend follow-up appointments and need routine tests (such as blood tests).

Is this an aspect of your child's care that you want to change or improve?
If yes, please provide a brief description of your current situation

ISUS appointments in [redacted] are on different days these need to be on same day to save on travel.
AND After noon appointments to give us time to get their

What works well at the moment with your child's follow-up appointments and routine tests?

[redacted] hospital is bette for us but not all care is available in their

What would you like to work better at the moment with your child's follow-up appointments and routine tests?

Blood test at home & always afternoon appointments.

How important is it to change/improve this aspect of your child's care?
Circle one option

High priority (circled) **Medium priority** **Low priority**

[illegible] your [illegible]

My overall aim would be to ...

HAVE [redacted] CARE UNDER ONE ROOF
WISH

Use this rating scale to show where you are now:

1. Very far from my / our aim	2.	3.	(4.)	5.	6.	7.	8.	9.	10. I have achieved my / our aim

Things I / we want to change / improve now.	How I / we want things to be	Care options and service provision my / our child / family need to get there?	Where is the **best** place for my / our child to be cared for in each situation / scenario	Potential imp[illegible] changes in m[illegible] child's cond[illegible] situation ove[illegible]
Nursing Care when [redacted] is very sick to the	in an ideal world all Care Tests Etc	This is being looked at for us	in our own Home he is very uncomfortable	
able to stay home & a Nurse to come to him	in the home	& discussions with Dr & CLC nurse	in hospital	

Planning ahead: [illegible] like to do with my child and family now or in the future

What would be your overall aim in changing or improving the activities that you do with your child and family now or plan to do in the future?

My overall aim would be to ...

Use this rating scale to show where you are now:

1. Very far from my / our aim — (2.) — 3. — 4. — 5. — 6. — 7. — 8. — 9. — 10. I have achieved my / our aim

Things I / we want to change / improve now.	How I / we want things to be	Care options and service provision my / our child / family need to get there?	Potential impact of changes in my / our child's condition / situation over time?
We would like more disable activities	with all of [redacted] needs a helper in the home	We need oxygen where we go soft cushions	
on the Island so that we could reach them easily.		wheel chair + medication so another pair of hands	
We would like to improve how we travel with [redacted]		~~[illegible]~~	
