# Supplementary material for: Planning ahead with children with life-limiting conditions and their families: development, implementation and evaluation of ‘My Choices’
Source: BMC Palliat Care. 2013 Feb 5;12:5. doi: 10.1186/1472-684X-12-5 (PMC3579717; doi:10.1186/1472-684X-12-5)
Supplement: Additional file 2 — parent booklet 2012. Blank booklet to download and use. [file 1472-684X-12-5-S2.pdf]

Choices for my Child

Booklet for Parents

ALL CLIPART IMAGES HAVE BEEN REMOVED FROM THIS BOOKLET TO AVOID COPYRIGHT INFRINGEMENT – PLEASE FEEL FREE TO PERSONALISE WITH YOUR OWN LOCAL ARTWORK

Introduction

What is this booklet for?

This booklet is to help you:

- Think about your child's care now and in the future
- Consider care choices and preferred locations of care in different scenarios
- Facilitate discussion with health care professionals
- Keep a record that can be added to over time

This booklet can be used in a number of ways:

If you want to you can:

- Take it home and use it in private,
- If you want to, fill it in with the support of health care professionals such as your doctor or nurse,
- Use it to help consider your thoughts and feelings,
- Use it to help talk through care choices and options with your children, family and health care professionals.

What care options and services are available?

When Filling in **'Choices for My Child'** – Please refer to the accompanying leaflet **'Types of Services and Important Terms'.**

Sometimes, not all care options and services will be available in your area – but knowing your preferred choice and preferred location of care will help the NHS to plan services for the future. Sometimes your care choices and preferred options may not be able to translate into actual services.

The ‘Choices for my Child’ booklet covers:

Thinking about and planning for different situations, including:

- **Caring for my child every day**
- **Activities I would like to do with my child and family**
- **Routine appointments and follow-up**
- **School**
- **Transition from children’s services to adult services**

And

‘What if?’ - scenarios, including:

- **If I or my family need a ‘short break’**
- **If my child is mildly unwell**
- **If my child is moderately unwell**
- **If my child is very unwell**
- **If my child may not recover**

Caring for my child every day at home.

Parents frequently need practical help and support to care for their children with complex health care needs and disabilities at home.

Is this an aspect of your Child's care that you want to change or improve?
If yes, please provide a brief description of your current situation

Do you have a 'key worker' to coordinate care? *Circle* **Yes No Don't know**

If you do not know ask your nurse or doctor to put you in touch

What works well at the moment with your child's everyday care?

What would you like to work better at the moment with your child's everyday care?

How important is it to change/improve this aspect of your child's care?
Circle one option

Very important **Important** **Not important**

Planning ahead: Caring for my child every day

What would be your overall aim in changing or improving your child's everyday care?

My overall aim would be to ...

Use this rating scale to show where you are now:

1. 2. 3. 4. 5. 6. 7. 8. 9. 10.

Very far from my/ our aim — I have achieved my/our aim

My ideas about improving or changing my child's everyday care

If appropriate, talk to your child and share your ideas with your nurse or doctor

Activities I would like to do with my child and family

Parents frequently have lots of activities that they would like to do now or in the future with their children

Is this an aspect of your child's care or family life that you want to change or improve? *Circle* **Yes No Don't know**

If yes, what would you like to change?

How important is it to change/improve this aspect of your child's care/ family life? *Circle one option*

Very important **Important** **Not important**

Planning ahead: Activities I would like to do with my child and family now or in the future

What would be your overall aim in changing or improving the activities that you do with your child and family now or plan to do in the future?

My overall aim would be to ...

Use this rating scale to show where you are now:

1.	2.	3.	4.	5.	6.	7.	8.	9.	10.
Very far from my/ our aim									I have achieved my/our aim

My ideas about planning for the future with my child and family

Follow-up appointments and routine tests

Children with complex healthcare needs and disabilities frequently attend follow-up appointments and routine tests (such as blood tests).

Is this an aspect of your child's care that you want to change or improve?

Circle **Yes** **No** **Don't know**

If yes, what would you like to change?

How important is it to change/improve this aspect of your child's care?
Circle one option

Very Important **Important** **Not important**

Planning ahead: Follow-up appointments and routine tests

What would be your overall aim in changing or improving your child's follow-up appointments and routine tests?

My overall aim would be to ...

Use this rating scale to show where you are now:

1.	2.	3.	4.	5.	6.	7.	8.	9.	10.
Very far from my/ our aim									I have achieved my/our aim

My ideas about changing or improving my child's follow-up appointments and routine tests

School

Your child may need practical help and support to get the best out of their schooling

Is this an aspect of your child's care that you want to change or improve?

Circle **Yes** **No** **Don't know**

If yes, what would you like to change?

How important is it to change/improve this aspect of your child's life?
Circle one option

Very important **Important** **Not important**

Planning ahead: My child's health care and school

What would be your overall aim in changing or improving your child's schooling?

My overall aim would be to ...

Use this rating scale to show where you are now:

1.	2.	3.	4.	5.	6.	7.	8.	9.	10.
Very far from my/ our aim									I have achieved my/our aim

My ideas about changing or improving my child's schooling

Transition to adult services

Children and young people with complex health care needs and disabilities transfer to adult services from around 16 years

Is this an aspect of your child's care that you have started thinking about, planning for, or been through? *Circle* **Yes** **No** **Don't Know**

If yes, please provide a brief description of your current situation

If you answered 'No' or Don't know' contact your nurse or doctor.

How important is it to change/improve this aspect of your child's care planning? *Circle one option*

Very important **Important** **Not important**

Planning ahead: Transition to adult services

If appropriate, what would be your overall aim in improving your child's transfer to adult services?

My overall aim would be to ...

Use this rating scale to show where you are now:

1. 2. 3. 4. 5. 6. 7. 8. 9. 10.

Very far from my/ our aim

I have achieved my/our aim

My ideas about improving my child's transition to adult services

What if ? scenarios

What if I / my family need a 'short break'

Parents and families frequently need 'short breaks' (formerly known as respite care) from their caring responsibilities

Is this an aspect of care that you want to change or improve?

Circle **Yes** **No** **Don't Know**

If yes, what would you like to change?

How important is it to change/improve this aspect of your care?
Circle one option

Very important **Important** **Not important**

Planning ahead: Short break care

What would be your overall aim in improving your short break care?

My overall aim would be to ...

Use this rating scale to show where you are now:

1.	2.	3.	4.	5.	6.	7.	8.	9.	10.
Very far from my/ our aim									I have achieved my/our aim

Ideas for my child and family's short breaks

What if my child is mildly unwell

When mildly unwell with illnesses such as coughs, colds, tummy bugs and urine infections etc – children usually need some extra care and a closer eye keeping on them until they are better

What works well when your child is mildly unwell?

What could work better when your child is mildly unwell?

How important is it to change/improve this aspect of your child's care?
Circle one option

Very important **Important** **Not important**

Planning ahead: If my child is mildly unwell

What would be your overall aim in improving your child's care if they are mildly unwell?

My overall aim would be to ...

Use this rating scale to show where you are now:

1.	2.	3.	4.	5.	6.	7.	8.	9.	10.
Very far from my/ our aim									I have achieved my/our aim

My ideas about my child's care when they are mildly unwell

What if my child is <u>moderately</u> unwell?

When <u>moderately</u> unwell – children usually need quite a bit of extra care, help with eating and drinking, sometimes more help with breathing such as additional nebulisers of physiotherapy, taking extra medicines, more frequent help with washing and hygiene and extra monitoring such as having someone sleep in the same room as them at night.

What works well when your child is <u>moderately</u> unwell?

What could work better when your child is <u>moderately</u> unwell?

How important is it to change/improve this aspect of your child's care?
Circle one option

Very important **Important** **Not important**

Planning ahead: If my child is moderately unwell

What would be your overall aim in improving your child's care if they are moderately unwell?

My overall aim would be to ...

Use this rating scale to show where you are now:

1. 2. 3. 4. 5. 6. 7. 8. 9. 10.

Very far from my/ our aim

I have achieved my/our aim

My ideas about my child's care if they are moderately unwell

What if my child is very unwell?

When very unwell – children require active treatment and care from doctors and nurses, sometimes in a hospital or in other places such as children's hospices, or your own home. They may need such things as an intravenous drip, intravenous medicines, help with breathing (such as oxygen and additional physiotherapy) and extra 24 hour care.

What works well when your child is very unwell?

What would you like to work better when your child is very unwell?

How important is it to change/improve/plan for this aspect of your child's care? *Circle one option*

Very important **Important** **Not important**

Planning ahead: If my child is very unwell

What would be your overall aim in planning for or managing your child's care if they are very unwell?

My overall aim would be to ...

Use this rating scale to show where you are now:

1.	2.	3.	4.	5.	6.	7.	8.	9.	10.
Very far from my/ our aim									I have achieved my/our aim

My ideas about if my child is very unwell

Planning ahead: what if doctors consider that your child may not recover?

How important is it for you and your family to have a clear plan in place for this aspect of your child's care?

Circle one option

Very important **Important** **Not important**

When would be an appropriate time for you and your family to start thinking about planning 'end of life' care?

Circle one option

Months in advance **Weeks in advance** **At the time**

If appropriate – what type of help and support would you and your family need to start thinking about and planning 'end of life' care with your child's care team?
Please describe:

Planning ahead: what if doctors consider that your child may not recover?

Would you and your family like to see a copy of an 'end of life' care plan template for future use?

Circle one option

Yes **No** **Don't know**

If 'Yes' contact your nurse or doctor.

You may not be ready to, or need to, consider care options – but if you have a preference – where would you prefer to care for your child:

- **Towards the end of their life?** Please state option:
- **At the end of their life?** Please state option:

Use this rating scale to show where you are now:

1. 2. 3. 4. 5. 6. 7. 8. 9. 10.

Very far from thinking about end of life care options

Very close to thinking about end of life care options

Other important things you wish to think about and plan ahead:

Notes:

This book was produced by Jane Noyes, Richard Hastings, Lucie Hobson, Ginny Bennett, Llinos Spencer and Richard Hain at Bangor University, on behalf of the 'My Choices' project team.

The 'My Choices' Project.
Contact:
Professor Jane Noyes
jane.noyes@bangor.ac.uk

Books in the 'My Choices' range include:

Book for children aged 6 – 10 years
Book for children aged 11 – 15 years
Book for young people aged 16 years and over
Booklet for Parents
Service Directory

Acknowledgements:

This booklet incorporates the philosophy of the 'Lifetime Framework' developed by Mary Lewis, Fiona Finlay and Simon Lenton, The Lifetime Service, Bath.

Aspects of the booklet design are based on a template developed by SPRU, University of York.

Cover artwork by Victoria Elizabeth Hulme ©

©Centre for Health-Related Research, Bangor University.

Funded by
Llywodraeth Cynulliad Cymru
Welsh Assembly Government
