# Supplementary material for: Planning ahead with children with life-limiting conditions and their families: development, implementation and evaluation of ‘My Choices’
Source: BMC Palliat Care. 2013 Feb 5;12:5. doi: 10.1186/1472-684X-12-5 (PMC3579717; doi:10.1186/1472-684X-12-5)
Supplement: Additional file 3 — My Choices Young person age 16+ 2012. Blank booklet to download and use. [file 1472-684X-12-5-S3.pdf]

My Choices

Booklet for young people aged 16+

ALL CLIPART IMAGES HAVE BEEN REMOVED FROM THIS BOOKLET TO AVOID COPYRIGHT INFRINGEMENT – PLEASE FEEL FREE TO PERSONALISE WITH YOUR OWN LOCAL ARTWORK

Introduction

What is this booklet for?

This booklet is to help you:

- Think about your care now and in the future
- Consider care choices and preferred locations of care in different scenarios
- Facilitate discussion with health care professionals
- Keep a record that can be added to over time

This booklet can be used in a number of ways:

If you want to you can:
- Take it home and use it in private,
- Fill it in with the support of health care professionals such as your doctor or nurse,
- Use it to help consider your thoughts and feelings,
- Use it to help talk through care choices and options with your friends, family and health care professionals.
- You can use this booklet to think about things, you don't have to fill it in.

What care options and services are available?

When using the **'My Choices' booklet** – You can refer to the accompanying leaflet **'Types of Services and Important Terms'.**

Sometimes, not all care options and services will be available in your area – but knowing your preferred choice and preferred location of care will help the NHS to plan services for the future. Sometimes your care choices and preferred options may not be able to translate into actual services.

The 'My Choices' booklet for young people covers:

Thinking about and planning for different situations, including:

- **Every day**
- **Social activities I would like to do, things I would like to achieve**
- **Routine appointments and follow-up**
- **Education/employment**
- **Transition to adult services**

And

'What if?' - scenarios, including:

- **If I or my family need a 'short break'**
- **If I am mildly unwell**
- **If I am moderately unwell**
- **If I am very unwell**
- **If I may not recover**

Everyday care at home

You may need practical help and support to care for yourself at home.

Is this an aspect of your care that you want to change or improve?
If yes, please provide a brief description of your current situation

Do you have a 'key worker' to coordinate care? *Circle* **Yes No Don't know**

If you answered 'No' or Don't know' ask your nurse or doctor.

What works well with your everyday care?

What would you like to work better at the moment with your everyday care?

How important is it to change/improve this aspect of your care?
Circle one option

Very important **Important** **Not important**

Planning ahead: My everyday care

What would be your overall aim in changing or improving your everyday care?

My overall aim would be to ...

Use this rating scale to show where you are now:

1.	2.	3.	4.	5.	6.	7.	8.	9.	10.
Very far from my aim									I have achieved my aim

My ideas about improving or changing my everyday care

Share these ideas with your parents, nurse and doctor

Social activities and things **I would like to achieve**

You may have lots of activities that you would like to do now or in the future

Is this an aspect of your care or family life that you want to change or improve? *Circle* **Yes No Don't know**

If yes, what would you like to change?

How important is it to change/improve this aspect of your care/ family life?
Circle one option

Very important **Important** **Not important**

Planning ahead: Social activities and things I would like to achieve now or in the future

What would be your overall aim in changing or improving the activities that you do with your family now or plan to do in the future?

My overall aim would be to ...

Use this rating scale to show where you are now:

1. 2. 3. 4. 5. 6. 7. 8. 9. 10.

Very far from my aim

I have achieved my aim

My ideas about planning and social activities

Follow-up appointments and routine tests

You may frequently attend follow-up appointments and routine tests (such as blood tests).

Is this an aspect of your care that you want to change or improve?

Circle **Yes** **No** **Don’t know**

If yes, what would you like to change?

How important is it to change/improve this aspect of your care?
Circle one option

Very Important **Important** **Not important**

Planning ahead: Follow-up appointments and routine tests

What would be your overall aim in changing or improving your follow-up appointments and routine tests?

My overall aim would be to ...

Use this rating scale to show where you are now:

1.	2.	3.	4.	5.	6.	7.	8.	9.	10.
Very far from my aim									I have achieved my aim

My ideas about changing or improving follow-up appointments and routine tests

Education/employment

You may need practical help and support to get the best out of your education and employment

Is this an aspect of your care that you want to change or improve?

Circle **Yes** **No** **Don't know**

If yes, what would you like to change?

How important is it to change/improve this aspect of your life?
Circle one option

Very important **Important** **Not important**

Planning ahead: My health care and education/ employment

What would be your overall aim in changing or improving your education/employment?

My overall aim would be to ...

Use this rating scale to show where you are now:

1.	2.	3.	4.	5.	6.	7.	8.	9.	10.
Very far from my aim									I have achieved my aim

My ideas about my health care and education/employment

Transition to adult services

You would usually transfer to adult services from around 16 years

Is this an aspect of your care that you have started planning for, or been through? *Circle* **Yes** **No** **Don't Know**

If yes, please provide a brief description of your current situation

If you answered 'No' or Don't know' ask your nurse or doctor.

How important is it to change/improve this aspect of your care planning?
Circle one option

Very important **Important** **Not important**

Planning ahead: Transition to adult services

What would be your overall aim in improving/planning for your transfer to adult services?

My overall aim would be to ...

Use this rating scale to show where you are now:

1. 2. 3. 4. 5. 6. 7. 8. 9. 10.

Very far from my aim — I have achieved my aim

My ideas about transition to adult services

What if ? scenarios

What if I / my family need a 'short break'

You may need a 'short break' from your parents/family, and your parents/ family may need a 'short break' if they have caring responsibilities

Is this an aspect of care that you want to change or improve?

Circle **Yes** **No** **Don't Know**

If yes, what would you like to change?

How important is it to change/improve this aspect of your care? *Circle one option*

Very important **Important** **Not important**

Planning ahead: Short break care

What would be your overall aim in improving/planning for if you need short break care?

My overall aim would be to ...

Use this rating scale to show where you are now:

1.	2.	3.	4.	5.	6.	7.	8.	9.	10.
Very far from my aim									I have achieved my aim

My ideas about short break care

What if I am mildly unwell

When mildly unwell with illnesses such as coughs, colds, tummy bugs and urine infections etc – young people usually need some extra care until they are better.

What works well when you are mildly unwell?

What could work better when you are mildly unwell?

How important is it to change/improve this aspect of your care?
Circle one option

Very important **Important** **Not important**

Planning ahead: If I am mildly unwell

What would be your overall aim in improving your care if you are mildly unwell?

My overall aim would be to ...

Use this rating scale to show where you are now:

1. 2. 3. 4. 5. 6. 7. 8. 9. 10.

Very far from my aim — I have achieved my aim

My ideas about if I am mildly unwell

What if I am moderately unwell?

When moderately unwell – young people usually need quite a bit of extra care, help with eating and drinking, sometimes more help with breathing such as additional nebulisers of physiotherapy, taking extra medicines, more frequent help with washing and hygiene and extra monitoring.

What works well when you are moderately unwell?

What could work better when you are moderately unwell?

How important is it to change/improve this aspect of your care?
Circle one option

Very important **Important** **Not important**

Planning ahead: If I am <u>moderately</u> unwell

What would be your overall aim in improving your care if you are <u>moderately</u> unwell?

My overall aim would be to ...

Use this rating scale to show where you are now:

1.	2.	3.	4.	5.	6.	7.	8.	9.	10.
Very far from my aim									I have achieved my aim

My ideas about if I am <u>moderately</u> unwell

What if I am very unwell?

When very unwell – young people require active treatment and care from doctors and nurses. They may need such things as an intravenous drip or naso-gastric feeding, intravenous medicines, help with breathing (such as oxygen and additional physiotherapy) and 24 hour sometimes one to one care.

What works well when you are very unwell?

What would you like to work better when you are very unwell?

How important is it to change/improve/plan for this aspect of your care?
Circle one option

Very important **Important** **Not important**

Planning ahead: If I am very unwell

What would be your overall aim in planning for or managing your care if you are very unwell?

My overall aim would be to ...

Use this rating scale to show where you are now:

1. 2. 3. 4. 5. 6. 7. 8. 9. 10.

Very far from my aim — I have achieved my aim

My ideas about if I am very unwell

Planning ahead: what if doctors consider that I may not recover?

How important is it for you to have a clear plan in place for this aspect of your care?

Circle one option

Very important **Important** **Not important**

When would be an appropriate time for you and your family to start thinking about planning care if doctors thought that you may not recover?

Circle one option

Months in advance **Weeks in advance** **At the time**

If appropriate – what type of help and support would you and your family need to start thinking about and planning this aspect of care with your care team?
Please describe:

Planning ahead: what if doctors consider that I may not recover?

Would you and your family like to see a copy of an 'end of life' care plan template for future use?

Circle one option

Yes **No** **Don't know**

If 'Yes' contact your nurse or doctor.

You may not be ready to, or need to, consider care options – but if you have a preference – where would you prefer to be cared for:

- **Towards the end of your life?** Please state option:
- **At the end of your life?** Please state option:

Use this rating scale to show where you are now:

1. 2. 3. 4. 5. 6. 7. 8. 9. 10.

Very far from thinking about end of life care options — Very close to thinking about end of life care options

Other important things you wish to think about and plan ahead:

Notes:

This book was produced by Jane Noyes, Richard Hastings, Lucie Hobson, Ginny Bennett, Llinos Spencer and Richard Hain at Bangor University, on behalf of the 'My Choices' project team.

The 'My Choices' Project.

Contact:
Professor Jane Noyes
jane.noyes@bangor.ac.uk

Books in the 'My Choices' range include:

Book for children aged 6 – 10 years
Book for children aged 11 – 15 years
Book for young people aged 16 years and over
Booklet for Parents
Service Directory

Acknowledgements:

This booklet incorporates the philosophy of the 'Lifetime Framework' developed by Mary Lewis, Fiona Finlay and Simon Lenton, The Lifetime Service, Bath.

Aspects of the booklet design are based on a template developed by SPRU, University of York.

Cover artwork by Victoria Elizabeth Hulme ©

©Centre for Health-Related Research, Bangor University.

Funded by
Llywodraeth Cynulliad Cymru
Welsh Assembly Government
