# Supplementary material for: Planning ahead with children with life-limiting conditions and their families: development, implementation and evaluation of ‘My Choices’
Source: BMC Palliat Care. 2013 Feb 5;12:5. doi: 10.1186/1472-684X-12-5 (PMC3579717; doi:10.1186/1472-684X-12-5)
Supplement: Additional file 4 — Children’s complex healthcare UK service directory 2012. Complex health key terms and directory for download and use. [file 1472-684X-12-5-S4.pdf]

What’s out there for children with complex health care needs?

Guide to services and key terms

This guide explains important terms, and describes the range and type of services for children with ‘palliative care’ needs

Contents

Key Terms **3**

Range and type of children's palliative care services **3**
Where does children's palliative care take place? *3*
How are children's palliative care services organized? *3*
What is complex care/continuing care? *5*
What are Direct Payments? *5*
What are the roles of children's community nurses? *5*
What is an 'employed carer'? *6*
What type of children's palliative care services do charities provide? *6*
What is short break provision (formerly known as respite care)? *6*
Specialist respite care. *7*
What is key working/care co-ordination? *7*
What is a children's hospice? *7*
What is hospice at home? *8*
What services do hospitals provide? *8*
What are end-of-life care services? *8*

Key terms

What is children's palliative care?

Palliative care for children and young people starts at diagnosis and is about making life experiences better for children and young people who are diagnosed with life-limiting and life-threatening conditions, and their families.

Palliative care focuses on quality of life for the child and support for the family and includes the management of symptoms, provision of family support and help with caring, short breaks, smooth transition to adult services and if appropriate care through death and bereavement.

What are Life-limiting conditions?

Life-limiting conditions are those for which there is no reasonable hope of cure and from which children or young people will die. Many young people live into adulthood with life-limiting conditions. Some of these conditions cause progressive deterioration, and the child or young person becomes increasingly dependent on parents and carers.

What are Life-threatening conditions?

Life-threatening conditions are those for which curative treatment may be feasible but can fail, such as cancer. Children in long-term remission or following successful curative treatment are not included.

Range and type of children's complex care services

Where does children's palliative care take place?

Depending on the service, children's palliative care can be provided in a variety of settings such as the family home, hospital, hospice or other locations. Many new services are designed to deliver care nearer to home.

How are children's palliative care services organized?

Children's Palliative Care Services are organized into 'universal' services available to all children, 'core' services which form the majority of children's palliative care services, and 'specialist' services when children need more specialist support.
See Figure 1.

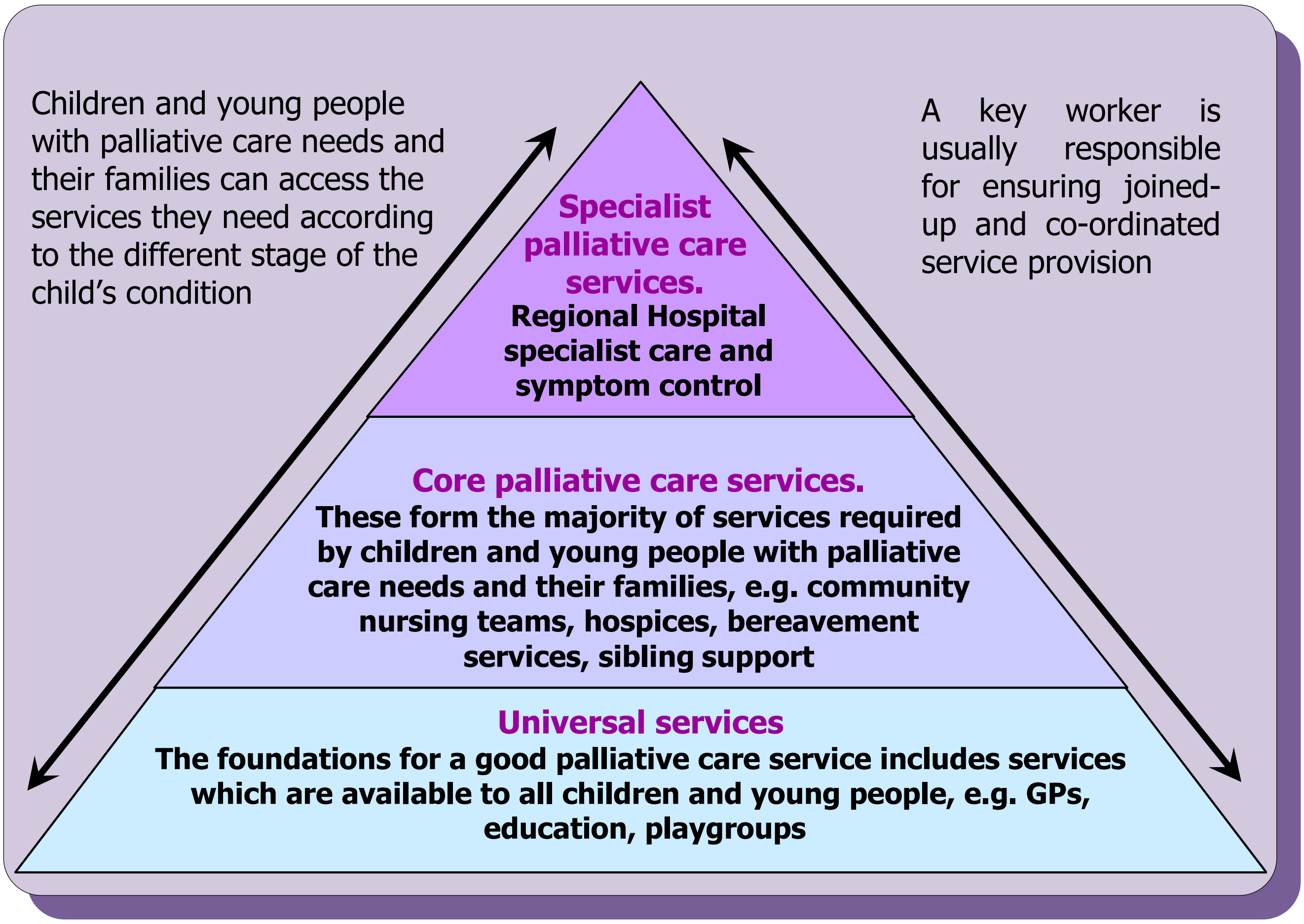

Figure 1: The organization of children's palliative care services – Adapted from 'Better Care, Better Lives, DH 2008

What count as 'universal', 'core', and 'specialist' services?

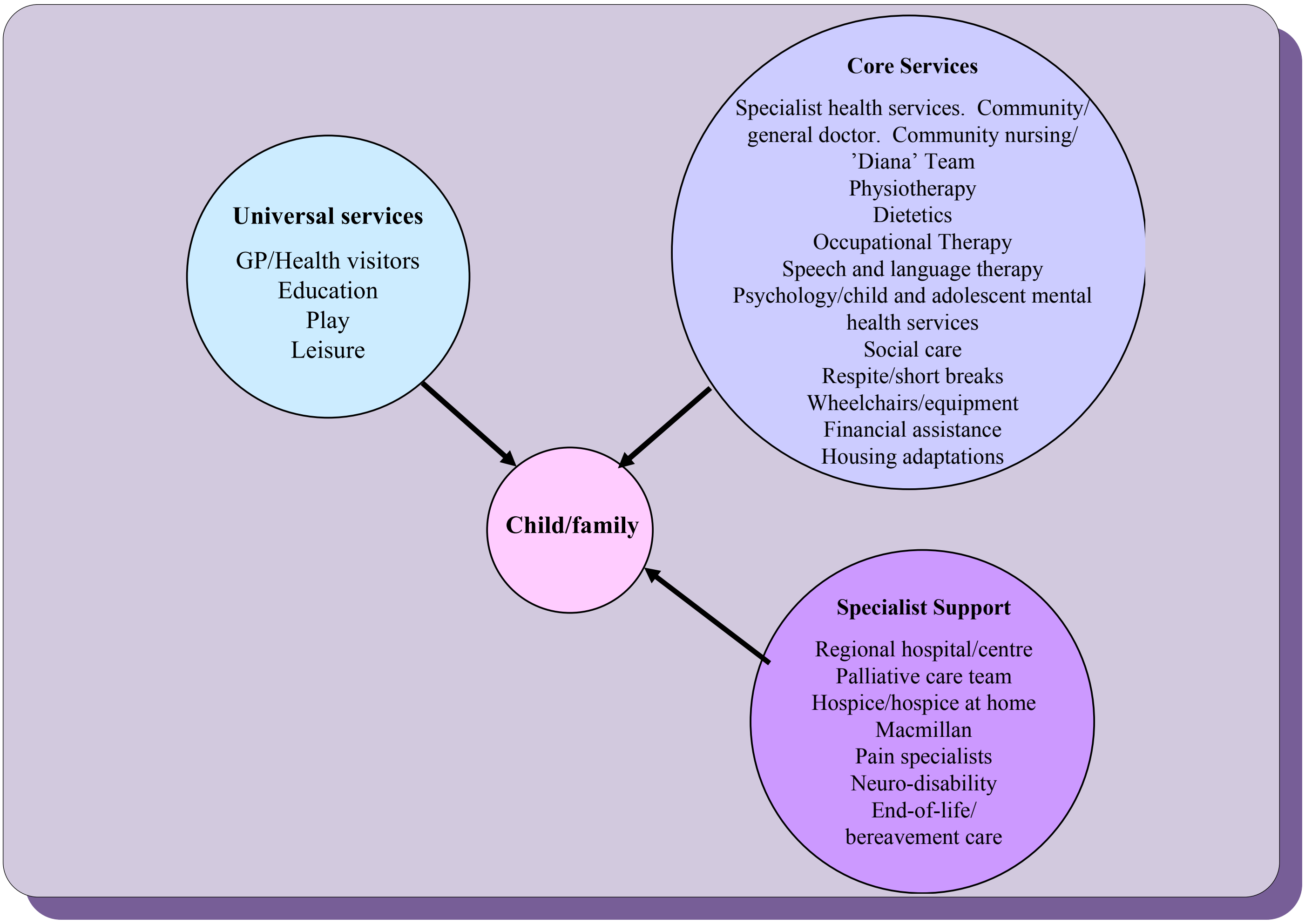

Figure 2: Types of 'universal', 'core' and 'specialist' services – Adapted from 'Better Care, Better Lives, DH 2008

What is complex care/continuing care?

'Continuing care' is a tailor made package of care beyond what is available through 'core', 'universal' and 'specialist' health services. It is provided to children with complex nursing care needs. Children and their families are referred for an assessment of continuing care need and if approved receive an individually tailored package of care. Assessments and re assessments can be 'fast tracked' for children who require additional care towards the end of their life.

What are 'Direct Payments'?

Direct payments are cash payments made to individuals who have been assessed as needing services, in lieu of social service provisions. By giving individuals money in lieu of social care services, people have greater choice and control over their lives, and are able to make their own decisions about how their care is delivered. They can be made to disabled people aged 16 or over, to people with parental responsibility for disabled children, and to carers aged 16 or over in respect of carer services.

The law has been changed so that it is a duty to make direct payments. This means that councils must make a direct payment to eligible individuals who are able to provide consent. Direct payments should be discussed as a first option at each assessment and each review.

What are the roles of children's community nurses?

There are several different roles of children's community nurses.

Generic children's community nurses work with all children who require home support (including short- and long-term follow-up and prevention of admission).

Specialist children's community teams (often multidisciplinary) work with children with specific or groups of conditions (e.g. children with disabilities, life-limiting conditions or malignant conditions).

Outreach children's community teams work with children who have been in hospital, to facilitate early discharge and prevent readmission.

Palliative care teams provide specialist care for the end of life.

Hospice outreach/hospice at home teams often provide respite care.

Home care support teams work with teams of carers providing care for children with very complex health needs.

Consultant nurses for children with complex needs often work with one or more teams of children's community nurses across geographical areas.

Clinical specialists work with children with specific conditions (e.g. diabetes, cardiac conditions), often sharing care with generic children's community nurses.

Community matrons focus on children with continuing health care needs to coordinate and streamline care.

Nurse-led clinics are run by the Children's community nurse in partnership with the GP or children's doctor.

Transitional care coordinators work with the increasing population of young people with complex needs who are surviving to adulthood and require support to assist transition to adult care or, as in many cases, ongoing care due to the lack of appropriate alternatives.

What is an 'employed carer'?

An employed 'carer' does not have a professional nurse training or qualification. They may be called a healthcare assistant, home support worker, home carer, family link carer, healthcare support worker and Crossroads worker.

They are trained to care for specific children in a safe and competent way while the child is well, following set instructions for all aspects of the child's care. Carers are trained to know when they have reached the limit of their role and when to seek help either from a parent or qualified nurse.

What type of complex care services do charities provide?

Examples of children's palliative care services provided by charities, include:

Hospice
Hospice at Home
Short breaks and 'respite care'
Funding for NHS staff such as children's community nurses
Employed and volunteer carers
Befriending and social support
Counselling
Advice and information
Equipment and aides
Leisure and entertainment
Meeting other children and families
Grants for equipment etc

What is short break provision (formerly known as respite care)?

Short breaks/respite care is care where the three main functions are:

- to provide the child or young person with an opportunity to enjoy social interaction and leisure facilities;
- to support the family in the care of their child in home or an alternative community environment such as a children's hospice; and
- to provide opportunities for siblings to have fun and receive support in their own right.

Short breaks/respite care may offer the whole family an opportunity to be together and to be supported in the care of their child, or it may offer care solely for the child or young person.

Examples of short break provision

Hospice/hospice at home – help families achieve a break together or time to themselves in a home-from-home environment, on in the family's own home.

Sitting services – people who regularly visit the child in his or her own home, enabling parents to spend time with their other children, have an evening out or even to do the shopping.

Befrienders/activity services – people who take the child out into the community, for example to the cinema, swimming, parks, shopping, and a wide variety of other activities.

Short break fostering – people who look after the child in the carer's own home, perhaps for one night, a weekend or longer, depending on the child's needs.

Community houses – where children and young people can have the opportunity to be creative with arts, crafts, cooking, baking and going out for activities within the community.

Domiciliary care – care provided at home which gives help with personal care and domestic tasks.

Specialist respite care

Specialist respite care refers to a setting of care, a programme of care or a service that provides additional services. It may take place in the child's home or in a setting outside of the home such as a hospital, long-term care facility or hospice. Specialist respite care provides the support required to meet the child's holistic care needs and enables children and families to access short break services. Specialist respite care will often address some aspects of symptom management.

What is key working / care co-ordination?

Key working/care co-ordination is a service involving two or more agencies, which provides disabled children and young people and their families with a system whereby services from different agencies are co-ordinated. It encompasses individual tailoring of services based on assessment of need, inter-agency collaboration at strategic and practice levels and a named key worker for the child and their family. Families with disabled children should only have a key worker if they want one (Care Co-ordination Network UK (CCNUK), 2006).

What is a children's hospice?

A children's hospice is an organisation which provides palliative care for a child or young person with a life-limiting condition and their family, and aims to meet all needs – physical, emotional, social and spiritual – through a range of services including children's palliative care, specialist respite care, terminal and emergency care, 24-hour telephone support, practical help, advice and information and bereavement support for all family members.

What is hospice at home?

Hospice at home is a term commonly used to describe a service which brings skilled, practical children's palliative care into the home environment. Hospice at home works in partnership with parents and families and provides hands-on expert nursing care, on a 24-hour basis, along with other elements of palliative care including:

- emotional, psychological and social support, counselling and spiritual care; access to specialist colleagues in other disciplines, such as physiotherapy, as required;
- provision of information, support, education and training where needed to all carers both lay and professional;
- close collaboration and communication with the primary care team, the child's acute hospital specialists if appropriate, and other agencies;
- specialist respite care;
- 24-hour end-of-life care; and bereavement support.

What services do hospitals provide?

Local and Regional hospitals services provide a range of out-patient and in-patient core and specialist services, including:

- Tests and investigations,
- Treatment,
- Symptom control,
- Follow-up clinics,
- Specialist community and outreach nurses, and
- End of life care.

What are end-of-life care services?

These are services that enable the supportive and end-of-life care needs of both child/ young person and family to be identified and met throughout the last phase of life and into bereavement. It includes management of pain and other symptoms and provision of psychological, social, spiritual and practical support. This is not confined to discrete specialist services but includes those services provided as an integral part of the practice of any health or social care professional in any setting.

Adapted from

© Association for Children's Palliative Care (ACT), *Children's Palliative Care: Descriptions and Definitions*

This book was produced by Jane Noyes, Richard Hastings, Lucie Hobson, Ginny Bennett, Llinos Spencer and Richard Hain at Bangor University, on behalf of the 'My Choices' project team.

The 'My Choices' Project.

Contact:
Professor Jane Noyes
jane.noyes@bangor.ac.uk

Books in the 'My Choices' range include:

Book for children aged 6 – 10 years
Book for children aged 11 – 15 years
Book for young people aged 16 years and over
Booklet for Parents
Service Directory

Acknowledgements:

This booklet incorporates the philosophy of the 'Lifetime Framework' developed by Mary Lewis, Fiona Finlay and Simon Lenton, The Lifetime Service, Bath.

Aspects of the booklet design are based on a template developed by SPRU, University of York.

Cover artwork by Victoria Elizabeth Hulme ©

©Centre for Health-Related Research, Bangor University.

Funded by
Llywodraeth Cynulliad Cymru
Welsh Assembly Government
