# Supplementary material for: Planning ahead with children with life-limiting conditions and their families: development, implementation and evaluation of ‘My Choices’
Source: BMC Palliat Care. 2013 Feb 5;12:5. doi: 10.1186/1472-684X-12-5 (PMC3579717; doi:10.1186/1472-684X-12-5)
Supplement: Additional file 6 — My Choices 6–10 years girl 2012. Blank booklet to download and use. [file 1472-684X-12-5-S6.pdf]

together for short lives

My Choices………..

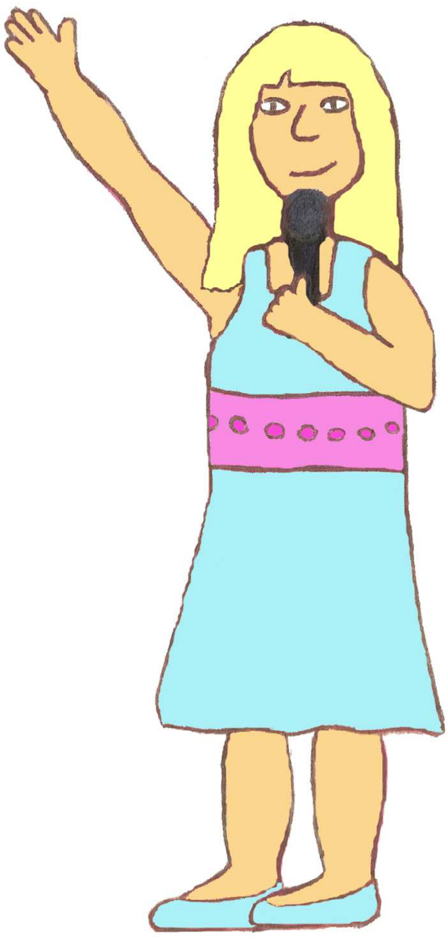

….About the care I need

This book belongs to….

ALL CLIPART IMAGES HAVE BEEN REMOVED FROM THIS BOOKLET TO AVOID COPYRIGHT INFRINGEMENT – PLEASE FEEL FREE TO PERSONALISE WITH YOUR OWN LOCAL ARTWORK

‘My Choices’

What is this book for?

This book is to help you:

- Think about your health care and what you want.
- Talk with your parents, family, friends and carers about what type of health care you want.
- Plan your health care with doctors, nurses and other people who look after you.

If you want to, you can ...

- Write down what is important to you.
- Add more information over time.

If you want to, ask an adult to help you fill in the book.

The ‘My Choices’ Book

The ‘My Choices’ book is to help you think about and plan your health care.

This book covers:

- Health care at home
- Fun things and future plans
- Health care at school

AND ‘What if’ situations, such as:

- What if my family need a short break?
- What if I am unwell?

All about me

My name is ____

I like to be called __________________________________

I live at ____

____

____

My birthday is on

____

These are the languages I speak

____

____

This is me and my family

You can draw a picture of yourself and your family here.

Who helps you to look after yourself?

(Tick the box next to the people who help you to look after yourself).

- ☐ Health Visitor
- ☐ Doctor
- ☐ Nurse
- ☐ Healthcare Assistant
- ☐ Physiotherapist
- ☐ Social Worker
- ☐ Psychologist
- ☐ Carer
- ☐ Other: ________________
- ☐ Other: ________________

My care at home

What is good and what is not so good about my health care at home. Write your thoughts in the bubbles.

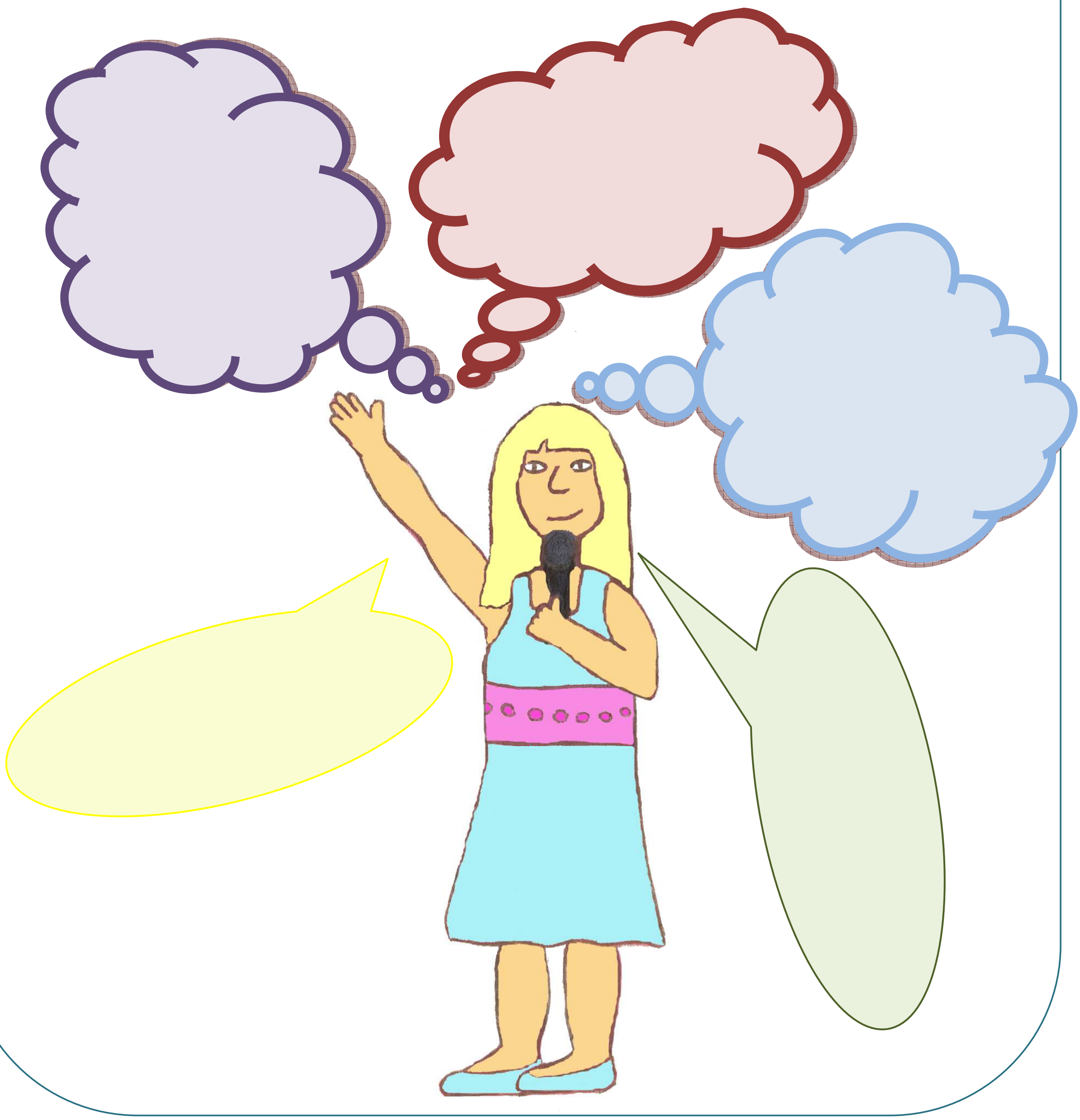

Fun things and future plans

I want to …………..

Write down all the things you want to do.

1.

2.

3.

4.

5.

6.

My care at school

What three things are good about your health care at school?
(Write them below)

1.

2.

3.

What three things are not so good about your health care at school?
(Write them below)

1.

2.

3.

What if…. My parents need a short break?

I would like to be looked after at the following places:

Circle all you choices

My Home

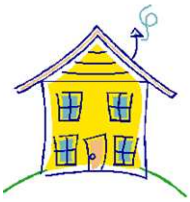

Hospice

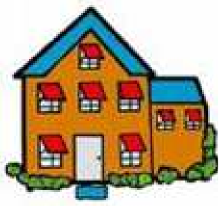

Hospital

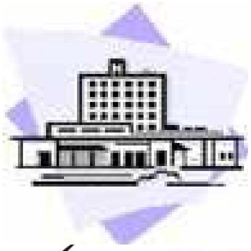

Relative, e.g. Grandma

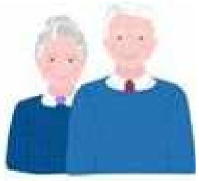

Somewhere else

?

Name: …………………

……………………………..

Somewhere else

?

Name: …………………

……………………………..

What if…. I am not well?

I would like to be looked after at the following places:

Circle all you choices

My Home

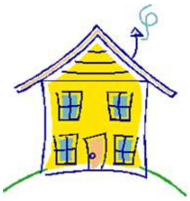

✓ ✗

Hospice

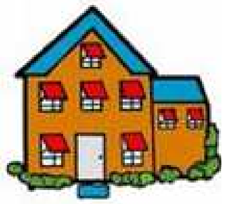

✓ ✗

Hospital

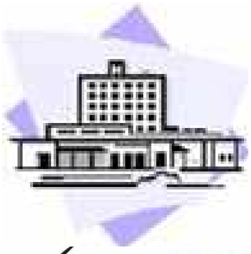

✓ ✗

Somewhere else

?

Name: …………………

……………………………..

✓ ✗

Somewhere else

?

Name: …………………

……………………………..

✓ ✗

Somewhere else

?

Name: …………………

……………………………..

✓ ✗

My choices for the future

Think of five things that you would like in the future to make your health and health care better. Write or draw your ideas in the stars below

1.

2.

3.

4.

5.

Anything else?

Is there anything else that you think is important?

This book was produced by Jane Noyes, Richard Hastings, Lucie Hobson, Ginny Bennett, Llinos Spencer and Richard Hain at Bangor University, on behalf of the 'My Choices' project team.

The 'My Choices' Project.

Contact:
Professor Jane Noyes
jane.noyes@bangor.ac.uk

Books in the 'My Choices' range include:

Book for children aged 6 – 10 years
Book for children aged 11 – 15 years
Book for young people aged 16 years and over
Booklet for Parents
Service Directory

Acknowledgements:

This booklet incorporates the philosophy of the 'Lifetime Framework' developed by Mary Lewis, Fiona Finlay and Simon Lenton, The Lifetime Service, Bath.

Aspects of the booklet design are based on a template developed by SPRU, University of York.

Cover artwork by Victoria Elizabeth Hulme ©

©Centre for Health-Related Research, Bangor University.

Funded by
Llywodraeth Cynulliad Cymru
Welsh Assembly Government
